# Different iPSC-derived neural stem cells shows various spectrums of spontaneous differentiation during long term cultivation

**DOI:** 10.3389/fnmol.2023.1037902

**Published:** 2023-05-02

**Authors:** Adelya Albertovna Galiakberova, Olga Igorevna Brovkina, Nikolay Vitalyevich Kondratyev, Alexander Sergeevich Artyuhov, Ekaterina Dmitrievna Momotyuk, Olga Nikolaevna Kulmukhametova, Alexey Aleksandrovich Lagunin, Boris Vladimirovich Shilov, Anton Dmitrievich Zadorozhny, Igor Sergeevitch Zakharov, Larisa Sergeevna Okorokova, Vera Evgenievna Golimbet, Erdem Bairovich Dashinimaev

**Affiliations:** ^1^Center for Precision Genome Editing and Genetic Technologies for Biomedicine, Pirogov Russian National Research Medical University, Moscow, Russia; ^2^Faculty of Biology, Lomonosov Moscow State University, Moscow, Russia; ^3^Federal Research and Clinical Center, Federal Medical-Biological Agency of Russia, Moscow, Russia; ^4^Mental Health Research Center, Moscow, Russia; ^5^Pirogov Russian National Research Medical University, Moscow, Russia; ^6^Department of Bioinformatics, Institute of Biomedical Chemistry, Moscow, Russia; ^7^Department of Bioinformatics, Koltzov Institute of Developmental Biology, Russian Academy of Sciences, Moscow, Russia; ^8^AcademGene LLC, Novosibirsk, Russia; ^9^Moscow Institute of Physics and Technology (State University), Dolgoprudny, Russia

**Keywords:** iPSC, neural stem cells, sc-RNA-seq, neural culture, differentiation

## Abstract

**Introduction:**

Culturing of human neural stem cells (NSCs) derived from induced pluripotent stem cells (iPSC) is a promising area of research, as these cells have the potential to treat a wide range of neurological, neurodegenerative and psychiatric diseases. However, the development of optimal protocols for the production and long-term culturing of NSCs remains a challenge. One of the most important aspects of this problem is to determine the stability of NSCs during long-term in vitro passaging. To address this problem, our study was aimed at investigating the spontaneous differentiation profile in different iPSC-derived human NSCs cultures during long-term cultivation using.

**Methods:**

Four different IPSC lines were used to generate NSC and spontaneously differentiated neural cultures using DUAL SMAD inhibition. These cells were analyzed at different passages using immunocytochemistry, qPCR, bulk transcriptomes and scRNA-seq.

**Results:**

We found that various NSC lines generate significantly different spectrums of differentiated neural cells, which can also change significantly during long-term cultivation *in vitro*.

**Discussion:**

Our results indicate that both internal (genetic and epigenetic) and external (conditions and duration of cultivation) factors influence the stability of NSCs. These results have important implications for the development of optimal NSCs culturing protocols and highlight the need to further investigate the factors influencing the stability of these cells *in vitro*.

## Introduction

Currently, neural stem cells (NSCs) and human neurons in culture *in vitro* are a hot topic in stem cell biology and biomedical research. In addition to the fact that *in vitro* studies of iPSC-derived NSC cultures provide opportunities to study the fundamental processes of neurogenesis, these cells can be used to model various genetically-inherited brain-related diseases (Kim et al., 2015; Bahmad et al., 2017; Dashinimaev et al., 2017). Also, these cells can provide a basis for the development of innovative technologies for the treatment of various neurodegenerative conditions, such as Alzheimer’s disease, Parkinson’s disease, amyotrophic lateral sclerosis, multiple sclerosis, multiple system atrophy, etc. (Garitaonandia et al., 2016; Bordoni et al., 2018). However, the main problems with this approach are in the choice of optimal protocols for obtaining such NSCs and in their subsequent cultivation. The use of iPSC-derived neural stem cells at the early stages has significant limitations associated with the presence of potential residual undifferentiated iPSCs that can lead to teratoma (Bulic-Jakus et al., 2016). Furthermore, long-term culturing of NSCs can lead to conditions unfavorable for transplantation, in which the cells become fragile and are less adaptable in the recipient’s body. Analysis of the transcriptomic profiles and the ratio of stem to mature cells in culture during passages will enable us to find the optimal period for safe and affordable transplantation.

Currently, as a general practice, pluripotent stem cells (PSCs) are used as the source of human NSCs. There are many different protocols for the differentiation of NSCs from PSCs (Galiakberova and Dashinimaev, 2020). Depending on the differentiation conditions, they produce NSCs with a diverse spectrum of differentiation into various types of neurons and glia (Watanabe et al., 2007; Chambers et al., 2009; Kim et al., 2011; Fedorova et al., 2019). For example, as a result of using some protocols, the obtained NSCs tend to differentiate into anterior brain neurons (telencephalic progenitors) (Watanabe et al., 2007; Shi et al., 2012), in others–into posterior or midbrain progenitors (Koch et al., 2009; Patani et al., 2009). In general, it can now be stated that with the main method used, the so-called Dual SMAD-inhibition method that uses small molecule inhibitors of SMAD signaling (TGFb-and BMP-signaling pathways), the resulting NSCs can be stably cultured until the 30th passage (Chambers et al., 2009; Li et al., 2011; Reinhardt et al., 2013).

It should be noted that neural cultures obtained from NSCs in this way, even with the use of specific growth factors, remain quite heterogeneous, given that NSCs can give rise not only to neurons of different types but also to glia (Chambers et al., 2009; Perriot et al., 2018). Moreover, it was shown that the ratio of these populations can depend on the conditions and duration of cultivation (Lam et al., 2019). Also it is assumed that iPSC lines derived from different donors or from different cell types have differences or limitations in differentiation spectra under the same conditions (Ohi et al., 2011; Kajiwara et al., 2012). This is probably due to differences in the lines at the epigenetic and genetic levels that emerged at the reprogramming stage (Koyanagi-Aoi et al., 2013; Zheng et al., 2013; Ma et al., 2014). Given these features, the question of the correlation between the spectrum range of NSCs differentiation potential and the initial lineage of iPSCs as well as the duration of cultivation remains open. Previously, it was shown that the physiological calcium activity of NSCs derived from PSCs changes significantly during 10 passages (Forostyak et al., 2013; Viero et al., 2014). At the same time, there are studies (Koch et al., 2009; Li et al., 2011) in which the authors postulate the development of protocols allowing to obtain and cultivate NSCs for 27 and 150 passages without significant changes in the morphological and immunocytochemical properties of cultures. However, a more detailed analysis of the composition of such neural cultures was not performed in these works. Here we took advantage of modern transcriptome analysis to identify and study cell types represented in heterogeneous neural cultures (Linnarsson and Teichmann, 2016).

In this study we investigated profile of spontaneous differentiation changes in several NSCs cultures obtained from different PSC lines with increasing duration of their passage in culture *in vitro*. For this purpose we utilized the most common protocol of NSCs production from PSCs using Dual SMAD inhibition to obtain a spectrum of spontaneously differentiated cells without additional guiding growth factors. The obtained neural cultures were studied to address the question whether the duration of *in vitro* passaging of the original NSCs affects the resulting neural cells.

## Materials and methods

### iPSC culture

iPS-KYOU, has been obtained in the Shinya Yamanaka laboratory (Kyoto University, Japan) by retroviral reprogramming of adult female skin fibroblasts. The iPS-KYOU cell line was purchased from the ATCC cell bank (KYOU-DXR0109B, ACS-1023™, ATCC®, United States).

iPS-AFS17, human Induced Pluripotent Stem Cells, obtained by lentiviral reprogramming of amniotic fluid stem cells by Dashinimaev et al. (2017).

iPS-DP human Induced Pluripotent Stem Cells, obtained by lentiviral reprogramming of dermal papilla cells (Muchkaeva et al., 2014).

iPS-DYP0730, human Induced Pluripotent Stem Cells, derived from dermal fibroblasts obtained from ATCC CCL-54 Detroit 532, a human Down syndrome cell line. The iPS-DYP0730 cell line was purchased from the ATCC cell bank (ACS-1003™, ATCC®, United States).

For all pluripotent stem cell passaging, we used ACCUTASE™ сell detachment solution (Stem Cell Technologies) and Rock-inhibitor Y-27632 (5 μM; Abcam), the plastic surface being pre-coated with Matrigel solution (1/40 in DMEM/F12) (Corning). The cells were cultured in mTeSR™1 medium (Stem Cell Technologies) at 37°C in a CO_2_-incubator with 5% CO_2_ and 100% humidity.

### Induction of neural differentiation of iPSCs

We directed the iPS cell lines into neural differentiation using the Dual SMAD inhibition method with a commercial Neural Induction Medium kit (PSC Neural Induction Medium, Life Technologies). After cultivating the cells for 4 passages, we obtained a homogeneous culture of neural stem cells, which we proliferated further on an NPM medium intended for culturing neural progenitor cells (DMEM/F12 (Capricorn) with DMEM (Capricorn), v1:1, N2 Supplement (Gibco), 20 ng/ml bFGF (Gibco), 20 ng/ml EGF (Gibco), Sodium Pyruvate (1 mM; Gibco), PenStrep (50 μg/ml; Gibco)) for further passages (up to 30). The NSС culture was subsequently made to spontaneously terminally differentiate at passages 5 (NSC-DP, NSC-DYP0730); 5, 10, 15, 20 and 25 (NSC-AFS17); 5, 10, 15, 20, 25, and 30 (NSC-KYOU). The terminal differentiation of neural stem cells was achieved using an N2B27 medium (Neurobasal (Gibco) with DMEM/F12 (Capricorn), v1:1, GlutaMax (1 mM; Gibco), Sodium Pyruvate (1 mM; Gibco), PenStrep (50 μg/ml; Gibco), β-Mercaptoethanol (0.1 mM; Sigma-Aldrich), N2-supplement (100x; Capricorn), B27-supplement (50x; Capricorn)) enriched nutrient medium without growth factors and with cell seeding on a pre-coated with Matrigel solution plastic surface at density, not more than 40*10^3^ cells/cm^2^. The cells were further cultured for 21 days. On the 14th day of differentiation, the neural cultures obtained from NSC were examined by RT-PCR and/or transcriptome analysis. In addition, 21 day neural cultures from the 5th (NSC-DP, NSC-DYP0730) or 5th and 25th (NSC-KYOU, NSC-AFS17) passages were also characterized by immunocytochemical analysis for the presence of neural markers. At each NSC passage, we counted the number of cells that had grown, and we always seeded the same number of cells (800*10^3^ cells per 6 cm Petri dish) in each passage. With these data, we plotted the proliferative rate curve, calculating the average number of population doublings in each passage as M = Log_2_(N_grown cells_/N_seeded cells_).

### Immunocytochemical staining

Before staining, the cells were washed with PBS and fixed for 15 min in 4% paraformaldehyde at room temperature (22°C–24°C). Then the cultures were gently washed in PBS three times (5 min at room temperature), incubated with the primary antibodies (Supplementary Table S1) in blocking solution (PBS with 10% FBS, 0.1% Tryton X-100 and 0.01%Tween-20) overnight (16–18 h) at +4°C, washed 3 times again and incubated with the secondary antibodies (Alexa Fluor 546 goat anti-mouse IgG or Alexa Fluor 488 donkey anti-rabbit IgG) (diluted 1:1000 in the blocking solution, Molecular Probes) for 1 h at 37°C. Then the cell nuclei were contrasted with DAPI (1 mg/ml in PBS). The images were obtained with an EVOS FL AUTO (Life Technologies) fluorescence microscope.

### Image analysis

All quantification procedures were made in the Fiji program. The process of image analysis was realized in the ImageJ macro language (Fiji-ImageJ 1.53 t version) (Rasband, 1997/2018; Schindelin et al., 2012). We have developed and used the integral estimation for each fluorescence channel. It included quantification of pixel brightness based on the histogram of the image. For increasing contribution of brightness, its value was squared for each pixel of image:


$$\mathrm{index of channel}=\sum \mathrm{pixel count}*{\mathrm{brightness}}^{2}$$

In the pairs of channels (red-blue and green-blue) was investigated a ratio index of antibody stained structures channel (red or green) to index of DAPI stained nuclei channel (blue). This ratio represents the level of antibody fluorescence. This process was automated with script written in the macro language of ImageJ. It was used to analyze 362 pairs of images of neural cultures. Each stained image was compared with a negative control for the secondary antibody (samples without the primary antibody). Results of image analysis include estimated ratio for each pair of images. R version 4.2.2 with the tidyverse (Wickham et al., 2019) and arsenal packages were used for statistical analysis and visualization. Analysis was done in two biological repeats (each had two technical repeats) separated over time.

### Total RNA extraction

Cells were harvested by Accutase (Stem Cell Technologies, Canada) and collected by centrifugation. Total RNA was extracted using the ExtractRNA kit (Evrogen, Russia), according to the manufacturer’s instructions. The RNA concentration was measured with an Implen P360 spectrophotometer system.

### Quantitative RT-PCR analysis

1 μg of total RNA was used to perform the cDNA synthesis. The first strand cDNA was synthesized using a Reverse transcriptase MMLV kit (Evrogen, Russia) with oligo(dT)-primers. Reverse transcription was performed for 1 h at 37°C and 10 min at 70°C to stop the reaction. Sequences of primers for the genes of interest are listed in Supplementary Table S2. We tested 6 genes previously described as neuron qPCR data normalization housekeeping genes (*ACTb*, *C1orf43*, *EMC7*, *GAPDH*, *PSMB4*, and *REEP5*; Artyukhov et al., 2017; Artyuhov et al., 2019) to find suitable normalization factors. According to geNorm software indications, *EMC7* and *PSMB4* were chosen for normalization. qRT-PCR was performed using the CFX96 PCR System (Bio-Rad Laboratories, Inc., United States). The temperature profile was (1) 95°C for 10 min, (2) 40 cycles of 95°C for 15 s and 60°C for 1 min, (3) melt curve analysis between 60°C and 95°C. HS-SYBR master mix (Evrogen, Russia) was used to prepare the reaction mixtures. Quantitative analysis was done in two biological replicates. The relative expression levels were assessed with the -ΔΔC_T_ method. We used Welch test (Student *t*-test for groups with different variances) to calculate value of ps, all statistical analyzes were performed using R software.

### Bulk RNA-seq

cDNA libraries were made starting with 1 μg of RNA with NEBNext Ultra II Directional RNA Library Prep (New England Biolabs, United States) and barcoded with index primers for pooled sequencing. The sequencing was performed to yield a median 20.9 million reads (range 11–34 million reads) of 2 × 150 base pairs per library. The gene-level counts were estimated with the “salmon” tool (version 1.2.1). The analysis for differentially expressed genes was performed with the “DESeq2” R package (version 1.30.1). Gene Set Enrichment Analysis was performed with “msigdbr” (version 7.4.1) and “clusterProfiler” (version 3.18.1) R packages with the default parameters on the list of genes, ordered by -log(*value of p*, BH-adjusted) ✕ sign(FC), where *p*-values and the FC (fold change of expression) were taken from the Deseq2 results. Gene ontology enrichment analysis of a list of common and co-directional differentially expressed genes in N-KYOU and N-AFS17 experiments was performed with the “enrichGO” function of the “clusterProfiler” package.

### scRNA-seq

#### Library construction

Neural cultures for analysis were harvested according to a standard protocol using Accutase (Gibco, United States). The cells were suspended in DPBS (PanEco, Russia) to use 12,800 cells in 12.8 μl DPBS per library preparation. We made libraries with 10x Genomics Chromium Single Cell 3’ Reagent Kit (Chromium Single Cell 3′ GEM, Library and Gel Bead Kit v3) according to the manufacturer’s instructions. We obtained 425 million reads in total (23,254 reads per cell).

#### Raw data processing

The quality filtration of reads was based on the following terms: the gene is expressed in at least 3 cells, the cell contains at least 300 different genes (min.cells = 3, min.features = 300). Further, we selected cells that contained fewer than 15,000 UMI and less than 10% mitochondrial RNA (nCount_RNA < 15,000 and percent.mt < 10). A Seurat package (version 3.1.1) in R software (version 3.6.3, R-Foundation, Vienna, Austria) was used to generate a gene-barcode matrix containing the barcoded cells and gene expression counts. The SCT transformation function from this package was used to normalize the molecular count data to the size of the library in each cell.

#### Clustering, visualization, and annotation

Cell-clustering and sub-clustering analyzes were performed with the FindClusters function of the Seurat package (resolution = 0.1). UMAP, an algorithm for dimension reduction, was applied for cluster manifold approximation and projection. The cell clusters were annotated based on the top-ranked DP genes from both the scRNAseq and RNAseq and from canonical neuronal markers from the literature.

#### Cell-cycle score analysis

We analyzed scores using the CellCycleScoring function of the Seurat package that stores S and G_2_/M scores in the object meta-data, along with the predicted classification of each cell in either the G_2_/M, S or G_1_ phases. The scores are based on the expression of the G_2_/M and S phase markers.

#### Pseudotime trajectory analysis

NSC differentiation was analyzed using the Monocle 3 package (version 0.2.3.0;^1^). Cells were organized into discontinuous trajectories along a trajectory corresponding to cell differentiation. The identified genes varied in their expression over these trajectories. A list of DE genes common for passages 5 and 25 related to the processes of cell proliferation, cell survival, cell growth, cell differentiation and cell migration were used to define a cell’s progress. Cluster 8 was selected as a null point for the Monocle tree structure.

#### Functional enrichment analysis

Gene set enrichment analysis (GSEA) was used to identify the gene set that was significantly enriched in the cluster. The Easy single cell analysis platform for enrichment (ESCAPE) and the ReactomeGSA package were the main instruments. During this step the data were aggregated to the gene set and pathway level according to the gene-level DE evidence. We downloaded all gene sets from the Molecular Signatures Database MSigDB,^2^ focusing on pathways related to neurons and stemness.

#### LD score regression

Stratified LD score regression, a method for partitioning heritability from GWAS summary statistics (Finucane et al., 2015), was applied to the top 200 specific genes for each of the 22 identified clusters in the scRNA-seq data. Specificity for genes was defined as the absolute log_2_FC produced as a result of the Seurat ‘FindMarkers’ function, applied to each of the clusters. GWAS summary statistics files were procured either from the United Kingdom Biobank (UKBB) or from the individual, respective GWAS studies (150 traits in total). We followed the LD score regression tutorial,^3^ with suggested default parameters.

## Results

### Neural cell cultures derived from different PSC lines show differences

**Figure 1:**
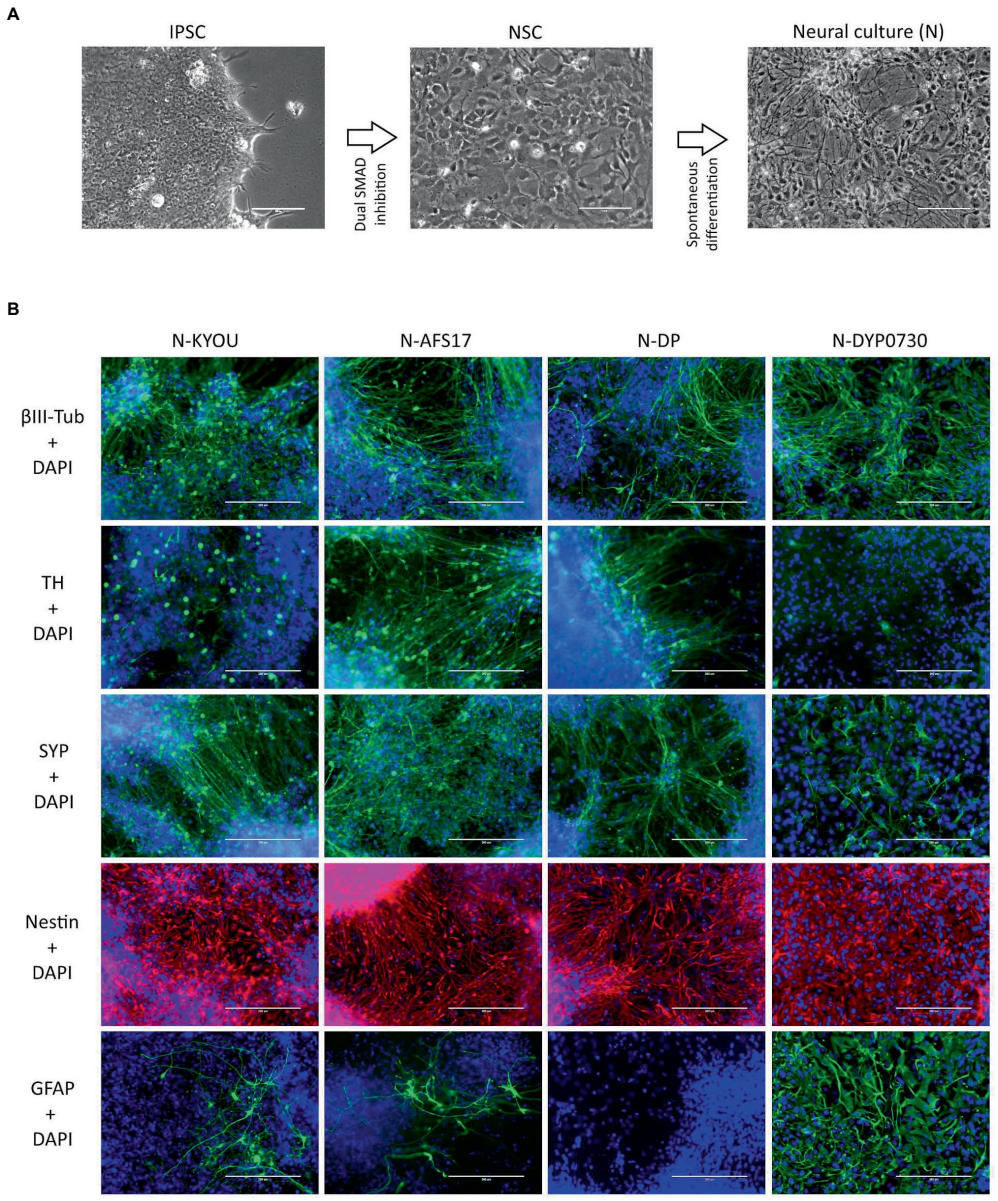
**(A)** Differentiation of IPSCs into neurons through the neural stem cell (NSC) stage using the DUAL SMAD inhibition protocol. IPSC–induced pluripotent stem cell; NSC–neural stem cell. Phase contrast microscopy, scale bar 200 μm. **(B)** Immunocytochemical staining of neural cultures (N) at day 21 of spontaneous differentiation of different NSC lines (KYOU, AFS17, DP and DYP0730) at 5 passages. The cells were stained for neuron markers b-III-tubulin, tyrosine hydroxylase (TH) and synaptophysin (SYP), neuron precursor cell markers nestin and glial cell marker GFAP. Fluorescence microscopy, scale bar 200 μm.

We tested the most popular protocol for obtaining NSCs from IPSCs, a protocol with Dual SMAD inhibition, on 4 different IPSC lines (iPS-KYOU, iPS-AFS17, iPS-DP and iPS-DYP0730) (Figure 1A). It should be noted that the iPS-DYP0730 line is derived from cells of a patient with Down syndrome and therefore has an extra 21 chromosomes. We expect that neural cultures derived from iPS-DYP0730 should be differ from cultures differentiated from the other lines (Mollo et al., 2021; Giffin-Rao et al., 2022) and therefore introduced this cell line into the analysis as a control for the differentiation spectrum shift. The moment when the cells developed an appropriate NSC morphology and were further cultured in NPM medium in the adherent state, we considered passage 0. From this moment, we cultured NSCs for 5 passages. At the 5th passage, NSC lines initiated spontaneous neuronal differentiation by eliminating growth factors from the medium. The thus-derived heterogeneous neural cultures of the 4 lines were maintained in N2B27 medium with 5 μM Rock-inhibitor. After 14–21 days of differentiation neural cultures (N-cultures) were analyzed by immunocytochemistry and quantitative RT-PCR.

While the NSCs were being cultured, most cells had the standard morphology for such cells, a rounded shape with short outgrowths, and with a high nuclear to cytoplasmic ratio (Supplementary Figures S1, S2). Upon initiation of these cells into spontaneous differentiation, we observed a typical change in culture morphology: the appearance of a significant number of cells each with a small soma and thin long neurites forming networks (Figure 1A; Supplementary Figures S1, S3), which we interpreted as the initiation of differentiation into neurons. However, it is important to note that both the NSC and N-cultures derived by spontaneous differentiation had a very heterogeneous cellular composition by morphology. For example, in the NSC cultures, we often found rosette-like structures of small cells with a distinct polarization (Supplementary Figures S1A,B; blue arrows), as well as regularly finding large flat cells with extensive cytoplasm (Supplementary Figures 1C,D; red arrows). In the neural cultures, although we identified most of the cells as neuron-like (Supplementary Figures 1E,F; green arrows), some cells also clearly retained NSC-like morphology (Supplementary Figures 1E,F; yellow arrows), and appeared to continue proliferation. Additionally, as in the NSC cultures, we observed flattened cells with an irregular shape, a large nucleus, without neurites, and with inclusions of vacuoles in the cytoplasm (Supplementary Figures 1E,F; red arrows).

**Figure 2:**
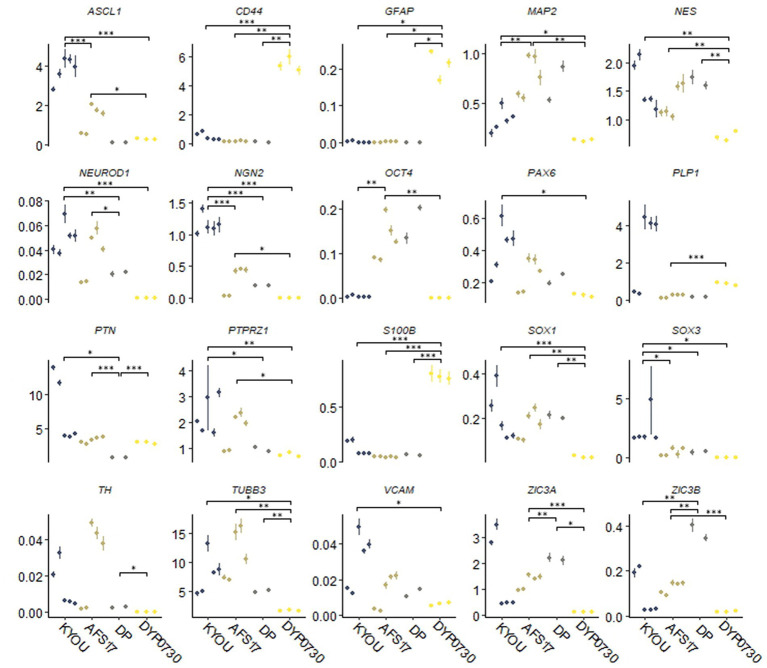
Results of quantitative RT-PCR analysis of marker gene expression levels in different neural cultures (N-KYOU, N-AFS17, N-DP, N-DYP0730) obtained from the corresponding NSCs at passage 5. On the ordinate axis is the expression level calculated by the−ΔΔС_t_ method, normalized to the expression of housekeeping genes. Each dot represents a biological replicate collected separately at a different time point (N-KYOU–5 replicates, N-AFS17–5 replicates, N-DP–2 replicates, N-DYP0730–3 replicates). The error bars indicate the standard deviation of the technical replicates, as determined by RT-qPCR. Asterisks indicate the statistical significance of the differences between the specified sample groups, calculated by the Welch test (*value of *p*<0.05, ** value of *p*<0.01, *** value of *p*<0.001), full statistics are available in Supplementary Table S10.

Moreover, the N-cultures obtained from different cell lines differed significantly from each other. In all neural cultures (N-KYOU, N-AFS17, N-DP and N-DYP0730) differentiated from the corresponding NSC lines at the 5th passages, markers of neural precursors–Nestin, Pax6 as well as neuron markers–b-III-tubulin, Synaptophysin (SYP) and tyrosine hydroxylase (TH) were detected (Figure 1B; Supplementary Figure 5E; Supplementary Table S3). However, the least TH-positive cells were found in N-DYP0730 (Supplementary Figure 5E; Supplementary Table S3).The glial cell marker, GFAP, was observed in all neural cultures except N-DP. However, cultures differed in the number of GFAP-positive cells (Supplementary Figure 5E; Supplementary Table S3). In N-KYOU and N-AFS17, 1–3 groups of GFAP^+^ cells were observed, whereas N-DYP0730 culture was significantly enriched in GFAP^+^ cells compared to the other cultures (Figure 1B). It is worth noting that b-III-tubulin^+^ cells in N-DYP0730 culture appeared to be rather heterogeneous in morphology: there were both neuron-like and NSC-like cells, and even cells whose morphology closely resembled GFAP^+^ cells of the same culture (Supplementary Figures S4A,C). Moreover, some Nestin^+^ cells had a similar morphology to GFAP^+^ cells (Supplementary Figures S4B,D). These observations are consistent with the fact that NSCs derived from iPSCs with trisomy 21 chromosome have an increased tendency to differentiate into astroglial cells (Mollo et al., 2021). Analysis of the expression levels of marker genes in four different neural cultures (N-KYOU, N-AFS17, N-DP and N-DYP0730) at passage 5 also showed significant variation between different cultures (Figure 2). Overall, we find a correlation between the data obtained by immunocytochemical staining and the measured gene levels. For example, neural cultures with chromosomal abnormality T21 (N-DYP0730), expressed levels of glial cell marker genes (*GFAP*, *CD44*, and *S100B*) much more and neuronal marker genes (*MAP2*, *NeuroD1*, and *TUBB3*) much less than the other three cultures. The expression of various marker genes (e.g., *ASCL1*, *MAP2*, *NGN2*, *OCT4*, *PTN*, and *SOX3*) also differed significantly between the three cultures with normal karyotype (N-KYOU, N-AFS17, N-DP), which seems to indicate a different cell composition produced by spontaneous differentiation.

**Figure 3:**
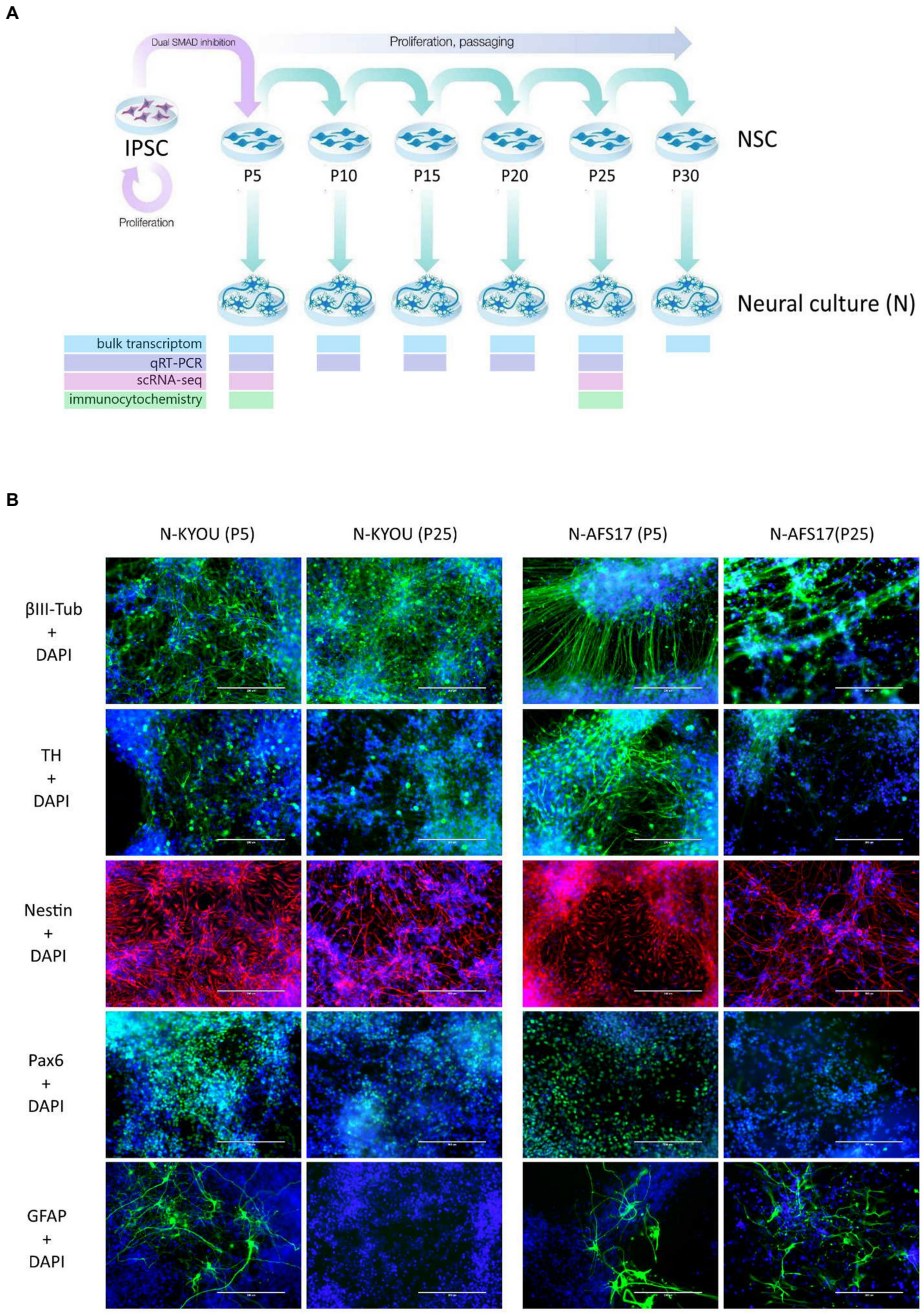
**(A)** General scheme of the experiment of investigate spontaneous differentiation of iPSCs into neurons. **(B)** Immunocytochemical staining of neural cultures at day 21 of spontaneous differentiation of N-KYOU and N-AFS17 at 5 and 25 passages (P). The cells were stained for neuron markers b-III-tubulin and tyrosine hydroxylase (TH), neuron precursor cell markers nestin and Pax6 and glial cell marker GFAP. Fluorescence microscopy, scale bar 200 μm.

### Neural cultures change with increasing passages of NSC cultivation

Next, we investigated how the duration of NSC cultivation affects their differentiation potential and the spectrum of neural cell types in cultures.

**Figure 4:**
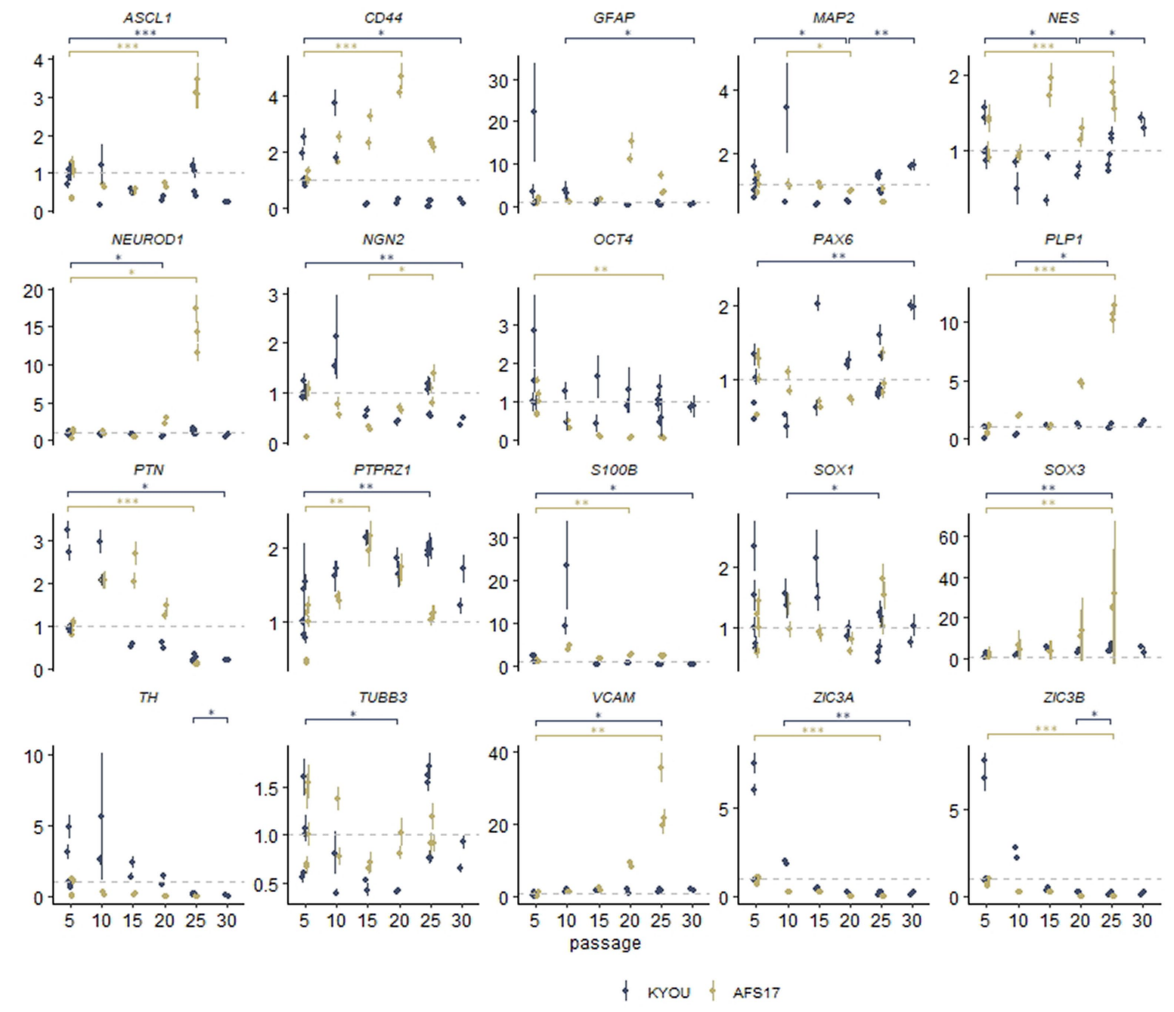
Results of quantitative RT-PCR analysis of the expression levels of marker genes in various neural cultures (N-KYOU and N-AFS17) obtained from the corresponding NSCs at passages 5, 10, 15, 20, 25, and 30 (for N-KYOU only). Along the y-axis is the expression level calculated by the−ΔΔCt method, normalized to the expression of housekeeping genes. Each dot represents a biological replicate collected separately at a different time point (N-KYOU p5–5 replicates, N-KYOU p10–2 replicates, N-KYOU p15–2 replicates, N-KYOU p20–2 replicates, N-KYOU p25–5 replicates, N-KYOU p30–2 replicates; N-AFS17 р5–5 replicates, N-AFS17 р10–2 replicates, N-AFS17 р15–2 replicates, N-AFS17 р20–2 replicates, N-AFS17 р25–3 replicates). The error bars indicate the standard deviation of the technical replicates, as determined by RT-qPCR. Asterisks indicate the statistical significance of the differences between the specified sample groups, calculated by the Welch test (* value of *p*<0.05, ** value of *p*<0.01, *** value of *p*<0.001), full statistics are available in Supplementary Table S10.

For this purpose, we continued to cultivate NSC-KYOU and NSC-AFS17 until the 30th passage. Every 5 passages some of the NSCs were initiated into spontaneous neural differentiation (Figure 3A). When NSC-KYOU and NSC-AFS17 were cultured from the 5th to the 30th passages for 169 days, we observed no significant changes in the culture proliferation rate (Supplementary Figure S6), and we observed no differences in the morphology of the cultured cells. During immunocytochemical staining of N-KYOU and N-AFS17 derived from NSC at passages 5 and 25 (N-cultures p5/p25), we observed the expected labeling of certain cell pools for markers of neural precursors (Nestin and Pax6) and neural markers (b-III-tubulin, SYP and TH) in both cases of both lines (Figure 3B; Supplementary Figures 5C,D; Supplementary Table S3). However, quantitative analysis of the staining results showed a decrease in the markers Nestin and Pax6 in N-cultures p25, compared to N-cultures p5 in both lines (KYOU and AFS17) (Supplementary Figures 5C,D; Supplementary Table S3). b-III-tubulin, although expressed at fairly high levels in N-cultures p5 and p25, has different dynamics in the two lines: in N-AFS17 p25 it decreases (Supplementary Figure 5C; Supplementary Table S3), while in N-KYOU p25, on the contrary, it increases (Supplementary Figure S5D; Supplementary Table S3). The change in the glial marker GFAP has an interesting dynamics. In N-KYOU p5 a small number of GFAP^+^-cells were present, but in N-KYOU p25 they were practically not detected (Supplementary Figure S5D; Supplementary Table S3). In the N-AFS17 p5, a small number of GFAP^+^-cells was also observed, but in N-AFS17 p25 their number significantly increased (Supplementary Figure S5C; Supplementary Table S3). The results of analysis of the expression levels of marker genes in N-KYOU and N-AFS17 neural cultures obtained at different passages confirmed significant differences either between both cultures at the same passage or between different passages in the same culture (Figure 4). The expression level of glial differentiation genes (*CD44*, *GFAP*, and *S100b*) shows a rapid decrease with passages in N-KYOU culture, whereas in N-AFS17 culture the expression of the same genes increases with passaging, which correlates with the result of immunocytochemical staining. We also observed a number of genes that tended to decrease expression with passaging in both cultures (*TH*, *PTN*, *ZIC3A*, and *ZIC3B*), while for some genes we were able to observe increased expression with passaging, such as *ASCL1*, *NEUROD1*, *PLP1*, *SOX3*, and VCAM (for N-AFS17) and *MAP2*, *NES*, *PAX6* (for N-KYOU).

**Figure 5:**
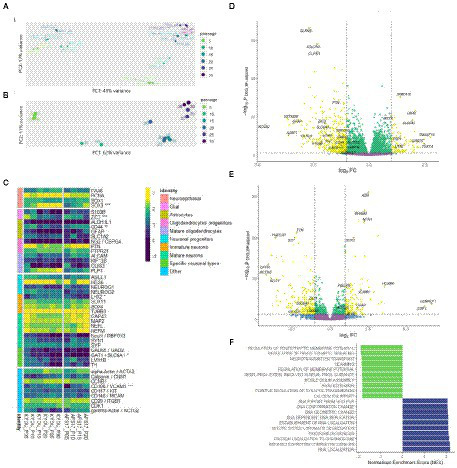
Bulk transcriptome analysis. **(A)** Principal component analysis applied to variance stabilized transformed expression data for both N-KYOU and N-ASF17 cultures. **(B)** Principal component analysis applied to variance stabilized transformed expression data for N-KYOU cultures. **(C)** Heatmap of change of expression in selected cell markers across passages for both N-KYOU and N-ASF17 cultures. Showing only genes that reached conventional statistical significance in DE analysis (“*” – *p* < 0.001; “**” – *p* < 1e-04; “***” – *p* < 1e-05). Color is transcripts per million (TPM) from highest (yellow) to lowest (blue) for each gene across the passages. The colored bar on the left indicates the functional annotation for a particular marker. The “Specific neuronal types” group includes markers of fully differentiated neurons: *GAD65* and *SLC6A1* for gabaergic, and *LMX1B* and *TH* for dopaminergic. The “Other” group includes markers for proliferating, stromal, endothelial or vascular cells. **(D,E)** Volcano plots of differentially expressed genes related to passage in the N-KYOU and N-ASF17 culture, respectively. Each dot represents a gene in coordinates of effect, log_2_ (fold change of expression at 30 passages vs. 5 passages) vs. statistical significance, −log_10_(*value of p*, BH adjusted). Yellow dots correspond to genes whose expression changes both abs(FC) > 2 and value of *p* <0.05. The yellow quadrants contain marked specific genes with high expression differences or significance, and known cell type markers The marker genes are *LHX2* (immature neurons), *RBFOX3* (NeuN) (mature neurons), *GRIN1* (glutamatergic neurons), *TH* (dopaminergic neurons), *GAD2* and *SLC6A1* (gabaergic neurons), *ALDH1L1, AQP4, CD44, GFAP* and *S100B* (glial), *PTN* (oligodendrocyte progenitors)*, PLP1* and *OLIG3* (mature oligodendrocytes), and *ACTG2* (vascular smooth muscle cells). **(F)** Results of gene set enrichment analysis (GSEA) for N-KYOU. The figure shows the top 10 depleted (green) and top 10 enriched (blue) GO BP terms sorted by normalized enrichment score (NES). All depicted terms reached statistical significance with *p* < 0.05 (BH-adjusted).

Thus, we successfully derived neural cell cultures spontaneously differentiated from NSCs in different passages during long-term cultivation. We observed a complex morphologically heterogeneous composition of these cell cultures consisting of many clearly different populations, the analysis of which was very difficult due to the limitations of fluorescence microscopy and qRT-PCR methods. Therefore, we proceeded to analyze the compositions of these cultures using transcriptomic data.

### Bulk transcriptome analysis of differences in the spectrum of spontaneous NSC differentiation during long-term cultivation

We collected samples of total RNA from the neural cultures derived from spontaneous NSC differentiation at different passages: 15 samples from 5th to 30th for NSC-KYOU and 8 samples from 5th to 20th for NSC-AFS17 (within 5-passage increments). RNA from each sample was processed to generate cDNA libraries for RNA-seq analysis. Principal component analysis of the transcriptomes in the case for the same N-KYOU neural culture at different passages shows that the number of passages is a major contributor to the differences, which can be clearly seen in the PCA plot (Figure 5B). Comparison of the mRNA expression spectra in NSC-KYOU-derived neural cultures of passage 30 and passage 5 showed multiple, significantly-differentially expressed transcripts associated with the number of passages (Figure 5D; Supplementary Table S4), and both up–and down-regulated expression. We performed gene ontology analyzes of such differentially expressed genes, and our results show that gene expression was depleted by the ontologies of neuron-specific genes and, conversely, enriched by the ontologies of genes involved in metabolic activity and cell proliferation in a passage-dependent manner (Figure 5F; Supplementary Table S5). We also found that the transcription of many specific genes used as markers for different cell populations changes with passage (Figure 5C). For example, as a rule, the expression of genes associated with mature neurons, specific neuronal types, and mature glial subtypes such as astrocytes and oligodendrocytes tended to decrease with the number of passages. At the same time, we observed an increase in gene expression typical of neuroepithelial and non-neural cells.

**Figure 6:**
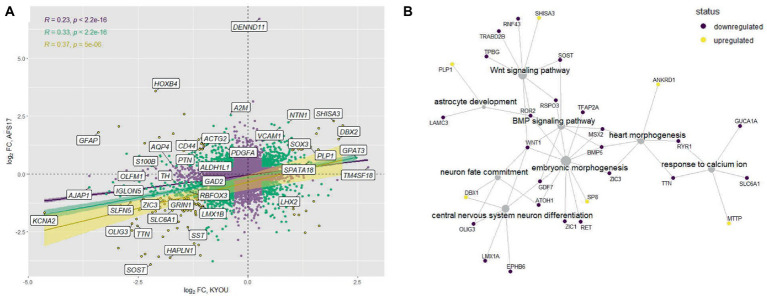
Joint analysis of DEG results for the two cell lines. **(A)** Comparison of DEG results for the two cell lines. Each point represents a gene in a log2 fold change coordinates for N-KYOU and N-AFS17 cultures. Yellow points represent genes with expression changes as big as abs(log_2_ fold change) > 1 in both experiments, green points -- abs(log_2_ fold change) > 0.33, violet points -- all other genes. Also shown are correlation coefficients, *p*-values for correlation tests (“pearson”) and linear fit models with 95% confidence level intervals for corresponded color-coded gene subset (violet for all genes, green for “violet” and “green” genes, yellow for “yellow genes only”). Specific genes are the same as specific genes on the volcano plots on Figures 2D,E. **(B)** Representative graph of gene ontologies and corresponding DEGs (*p-level* < 0.05) in both experiments with matched directions of effect. Yellow nodes denote genes with expression rising with number of passages, dark purple nodes represent genes with expression falling with number of passages.

Comparison of the complete transcriptomic data of N-KYOU and N-AFS17 shows that the cultures are different. The PCA plot shows that the N-cultures obtained from different passages of NSC-AFS17 are grouped separately from those from NSC-KYOU. It should be noted that the main source of variation in gene expression is the difference in the initial iPSC cell lines. Nevertheless, the second major component agrees well with the number of passages for both cell lines (Figure 5A). This may imply that there are general trends in culture variation with increasing duration of NSC cultivation. RNA-seq analysis of the N-AFS17 revealed relatively fewer differentially expressed genes than in the case of N-KYOU, without significant GSEA enrichment, probably due to a smaller sample (Figure 5E; Supplementary Table S6). The results of comparison of changes in gene expression in N-AFS17 with the N-KYOU culture are presented on Figure 6A (Supplementary Figure S13). While there are a number of genes with opposite directions of effect (most notably, *GFAP*), effects in both experiments are in general weakly but significantly correlated, *R* = 0.23 for all genes, *R* = 0.37 for genes with *p-level* < 0.05 (in both experiments). There are 8 upregulated (for example, *SHISA3* and *PLP1*) and 59 downregulated DEGs with abs(FC) > 2 and adjusted *p-level* < 0.05 and with matched directions of effect. GO enrichment analysis of these genes in both experiments reveals enrichments in 42 gene ontologies (Figure 6B; Supplementary Figure S7; Supplementary Table S7). Notable enriched ontologies are the BMP (adjusted value of *p* = 0.0078) and WNT (adjusted value of *p* = 0.027) signaling pathways.

Thus, we obtained data indicating that the composition of the neural cell culture changes with the number of NSC passages, which prompted us to conduct experiments to study the composition of the heterogeneous cell populations at the level of individual cells.

### Single-cell RNA-seq analysis of KYOU neural cultures during long-term cultivation

To prove that long-term cultivation of NSCs leads to changes in cell populations in the neural cultures differentiated from them rather than to changes in the overall transcriptomic profile, and to confirm the heterogeneity of neural cultures obtained from iPSCs using Dual SMAD–we performed scRNA-seq analysis for neural cultures obtained from NSC-KYOU from passages 5 and 25. To create single cell cDNA libraries, we used cells from two samples: 5th and 25th passages, using two independent biological replicates for each passage. After quality filtering, 8,105 cells from passage 5 and 6,341 cells from passage 25 were included in the data analysis.

**Figure 7:**
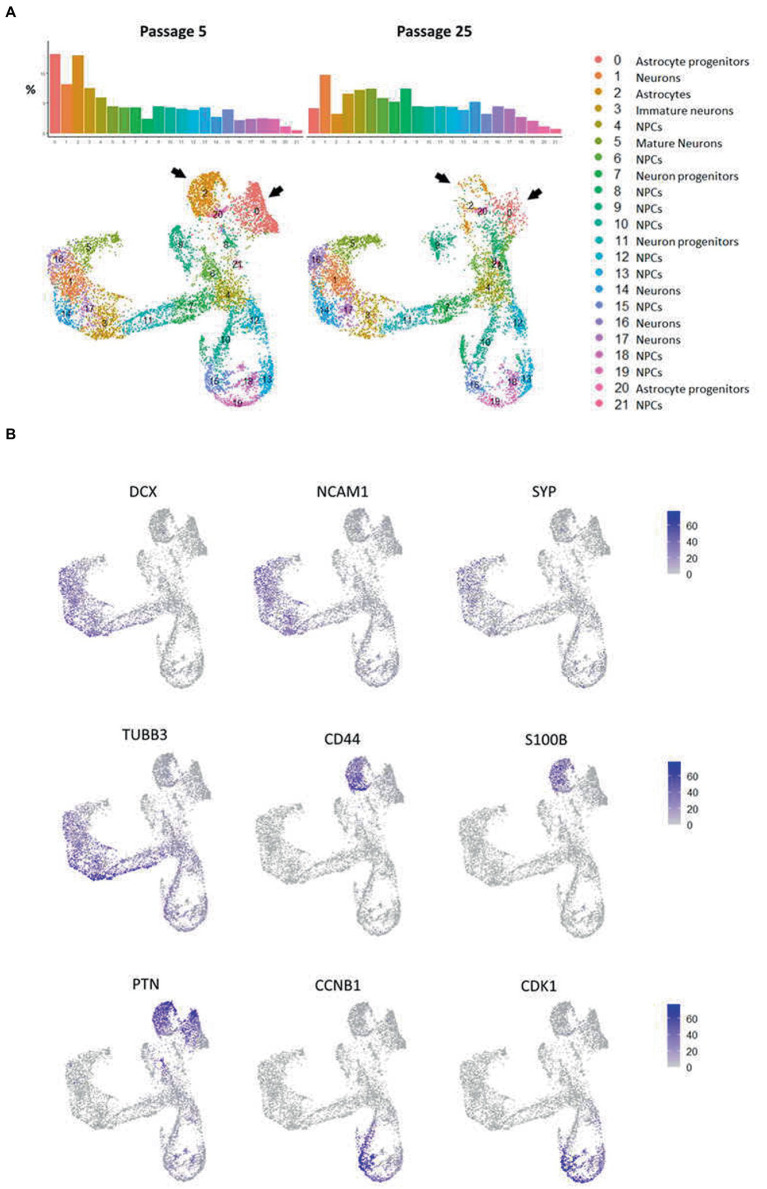
Cluster partition and annotation of scRNAseq data from N-KYOU p5 and p25. **(A)** 22 clusters revealed by the UMAP dimensional reduction of the scRNAseq algorithm in N-KYOU p5 and p25. The black arrows indicate the two clusters that differ most significantly in N-KYOU p25 compared to p5. **(B)** Gene activity of specific genes across the same UMAP space as on panel A. Markers of typical neural cell populations and cell cycle markers.

The primary analysis showed that both cell cultures were highly heterogeneous. According to the automated SingleR annotator, 21 distinct cell clusters/populations were identified with characteristic genes listed in Supplementary Table S8. We defined most of them as neural progenitor cells (NPCs), the remaining clusters were defined as astrocytes, astrocyte progenitors, mature neurons, neurons and neuronal progenitors (Figures 7A,B; Supplementary Figure S8). When comparing the distribution of clusters between the two passages, we observed a dramatic decrease in the representation of clusters 0 and 2, in N-KYOU p25 compared to N-KYOU p5 (Figure 7A; black arrows). We identified these clusters as astrocytes and astrocyte progenitors, because cells from those clusters express distinct glial markers, which explains their identification as DE genes in bulk transcriptome analysis (Figure 5C). The most characteristic markers for cluster 0 were *SCL6A1* (a gene for GABA transporter GAT1 and a marker for inhibitory neurons, astrocytes and oligodendrocytes), as well as for *SPARCL1* (a gene for the extracellular matrix Ca^2+^-binding protein, expressed by astrocytes) (Minelli et al., 1995; Kucukdereli et al., 2011). Markers for cluster 2 were genes *S100B* (gene for S100 Calcium Binding Protein B, a marker of oligodendrocytes and astrocytes), *CLU* (gene for clusterin, a marker of Muller glia and to a lesser extent astrocytes) and a gene for transcription factor ZIC3 (major marker for Muller glia and also for microglia and astrocytes). Also, cluster 2 was characterized by distinct expression of the hyaluronic acid receptor gene *CD44* and the Pleiotrophin gene (*PTN*), which are also implicit markers of astrocytes (Figure 7B) (Yeh et al., 1998; Liu et al., 2004; Cai et al., 2012; Dzwonek and Wilczynski, 2015; Linnerbauer et al., 2022). Interestingly, the neighboring small glial cluster 20 which is the only *TAGLN^+^* cluster (a gene for transgelin, a smooth muscle cell marker) remained almost unchanged with passages in terms of cell representation, but became more actively proliferating as the proportion of cells in phases S and G_2_ increased in N-KYOU p25. A similar situation is reflected in the expression of many genes, characteristic for this cluster, which were also identified in our bulk transcriptome analysis.

The representation of the clusters we defined as neurons on different stages of maturation (5, 16, 1, 17, and 14) slightly increased in passage 25 compared to passage 5. Also worth noting, was a significant increase in the representation of clusters 6 and 8, in N-KYOU p25 compared to p5.

**Figure 8:**
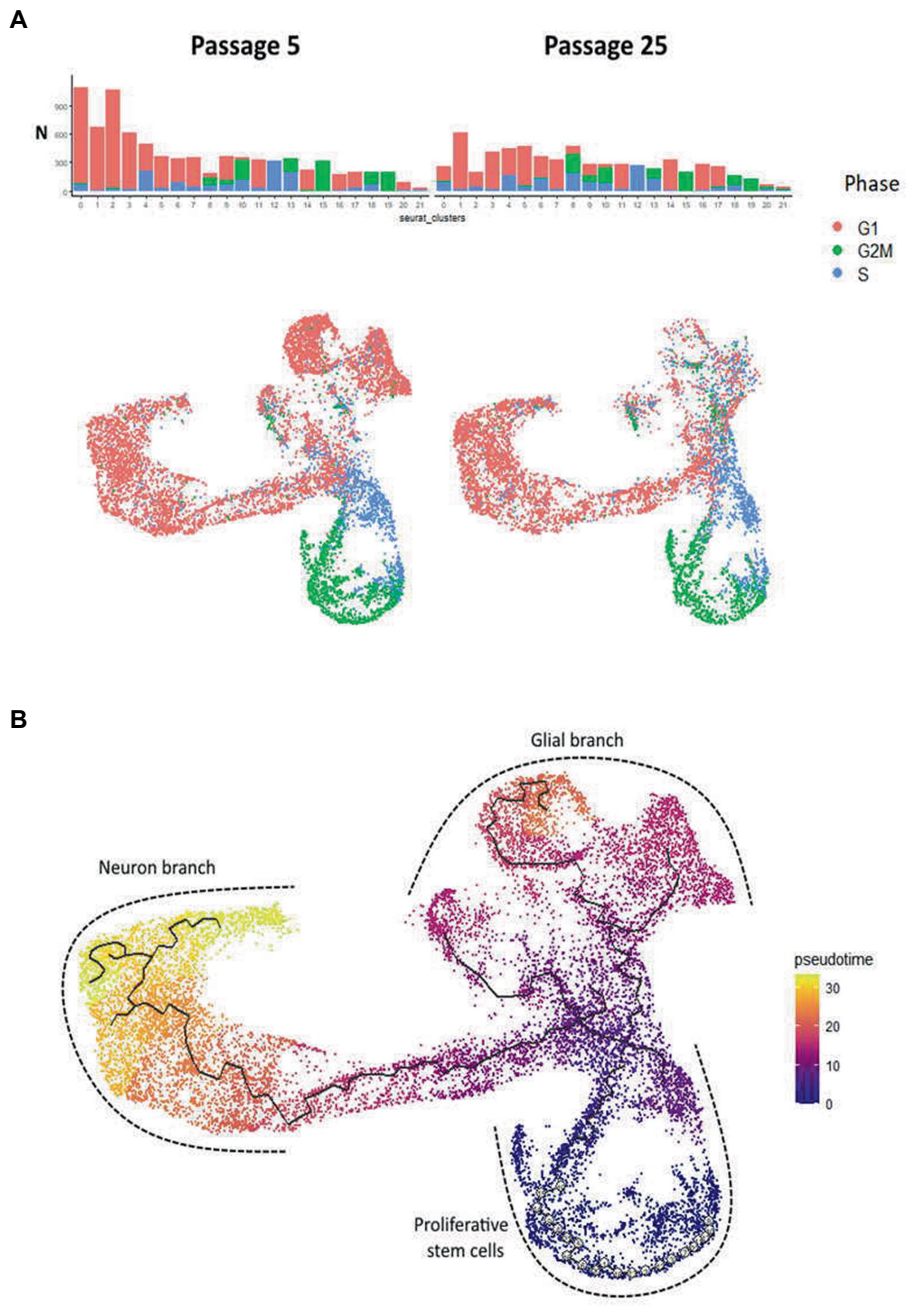
Cell cycle phases and pseudotime analysis of scRNAseq results from N-KYOU p5 and p25. **(A)** The occupancy and cell cycle of the detected clusters, *N* indicates the number of cells in the cluster. **(B)** Monocle trajectory analysis shows several directions of stem cell differentiation, from the area of proliferative stem cells through the glial or neuron branches.

Analysis of proliferative cycle markers in the clusters showed that the cell populations were clearly divided by cell cycle phases (Figure 8A). In N-KYOU p5, we found 13 clusters in which more than 80% of the cells were in the early G_1_ phase. In N-KYOU p25, the number of such clusters was reduced to 11. Because the G_1_ phase is the start for the G_0_ phase, a non-proliferative stage of the cell cycle, we considered the cells in G_1_ to be terminally differentiating cells. Correspondingly, we regarded the cells determined in the G_2_/M and S phases as actively dividing progenitor and stem cells. This allowed us to define the group of cell clusters located in the lower part of the UMAP (10, 12, 13, 15, 18, and 19) simply as cycling neural stem or progenitor cells.

According to the Monocle trajectory analysis, it became clear that there are several directions of stem cell differentiation whose bifurcation point is located in cluster 4. Cluster 4 which is at the crossing of cycling cell clusters (lower branch) and is further divided into two global cluster groups, “left” group (3, 17, 14, 1, 16, and 5) and “upper” group (9, 20, 0, 2; Figure 8B). The “left” clusters group is characterized by the expression of neuron-specific genes: *STMN2*, *DCX*, *GAP43*, *TUBB3*, *SYP*, *BASP1*, *RTN1*, *NCAM1*, *SOX3*, and SOX11, which supports our hypothesis that this branch of differentiation leads to mature neurons (cluster 5) (Supplementary Figure S9). Clusters 7 and 11, which are on the pathway to the “left” clusters, also contain stem markers (*ASCL1*, *SOX3*, *PAX6*, *NEST*, *POU3F2*, *SOX1*, and *NEUROG2*) along with neuronal markers and are therefore identified as early neuronal progenitors (Supplementary Figure S10). By the expression of astroglia-specific genes and their precursors (*S100B*, *CD44*, *ITGA6*, and *CLU*), we confirmed the “upper” clusters group as a glial branch of differentiation (Supplementary Figure S11).

**Figure 9:**
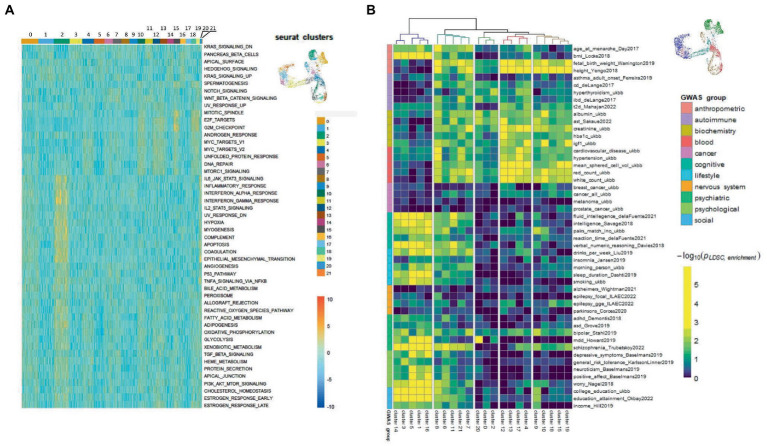
Functional and genetic signatures for identified cell clusters in the scRNA-seq data from N-KYOU p5 and p25. **(A)** Functional interpretation of a list of genes, specific for each cluster. Heatmap of top clustered Hallmark ontologies is shown. Columns represent scRNA-seq cell clusters, colored as in bar on the right with UMAP picture as a reference. **(B)** Heatmap of representative LDSC results for each cluster. Heatmap color is LDSC enrichment [−log_10_ (*p*-values), *p*-values are BH adjusted]. Rows are traits grouped in 11 categories. Columns are scRNA-seq cell clusters. Only 50 of the most enriched traits in every category are shown due to lack of drawing space; the whole data are present in Supplementary Table S9. Colors on the UMAP reference picture are the same as those of the cluster branches on top of the heatmap and correspond to five major groups, defined in the LDSC data.

To further characterize the clusters we performed functional enrichment analysis with the top results presented in Figure 9A. The most enriched pathways were those related to cell cycle progression for “stem” clusters and the number of apoptotic pathways for cluster 2. Furthermore, we applied LD score regression to the cluster-specific genes to understand how such cell clusters are related to 150 human heritable traits (Figure 9B; Supplementary Table S9). We found that, as expected, the greatest number of “neural” clusters (1, 3, 5, 14, 16, and 17) are enriched in genetic signals for the most cognitive, psychiatric (especially for schizophrenia), psychological, educational traits, and for BMI, but not for neurological and neurodegenerative diseases. The same is true for clusters 6, 7, 8, 11 and 21–but to a lesser extent, which can be explained in that these cells are generally less mature neural cells. On the other hand, the “glial” clusters (0, 2, 20) are mostly depleted in any of the tested features. A notable exception is the unexpected enrichment for major depressive disorder seen in cluster 20 (*p*, BH adjusted = 0.0013). Two groups, clusters 4, 12, 13, 17 and clusters 9, 10, 15, 18, 19 have very similar LDSC enrichment patterns. They are all enriched in traits for some anthropometric, blood and biochemical markers and all of them, with the exception of cluster 17, were identified as neuronal stem cells.

Interestingly, the DEGs associated with WNT and BMP signaling pathways, which were detected by bulk transcriptomes analysis of N-KYOU and N-AFS17 at early and late passages, have cell type-specific localization according to our N-KYOU scRNA-seq data. As expected, the downregulated genes are localized in the glial “upstream” clusters 0 (*GDF7*, *RSPO3*, *TPBG*, *WNT1*) and 2 (*BMP5*, *MSX2*, and *RNF43*), as well as in the “downstream” cluster of cyclic neural stem cells (*MSX2*, *RNF43*, *RSPO3*, and TPBG), decreasing with transition. The only elevated *SHISA3* gene is also localized in the “lower” clusters (Supplementary Figure S12).

As our pseudo-time analysis suggests, cells from cluster 20 are precursors for cluster 2 cells, so the changes of gene programs in this cluster are probably responsible for the observed cluster 2 depletion in N-KYOU p25. Interestingly the top LDSC signals for specific genes of cluster 20 were the significant enrichment of those for aspartate aminotransferase (AST) blood levels and major depression disorder (MDD) (Figure 9B), which differentiate this cluster from similar clusters 0 and 2 in this analysis. This could be important, because AST, normally a biomarker for liver health, has also been found to be a marker for MDD (Lee, 2021), thus suggesting that this particular effect is localized in similar cells in the human body. The same pattern is seen for cluster 8, which is upstream of clusters 0 and 20. As passages were increased from passage 5 to 25, its representation increased 3-fold (from 2.5 to 7.5%), with most of the cluster cells being in the G_2_M and S phases. This indicates that it appears to be a constantly dividing population of neural progenitors, committing to differentiation in the glial direction, but which loses the ability to fully differentiate as it passes 25 passages (more than 130 days of cultivation and more than 70 average cycles of division).

More than half of the clusters in N-KYOU p5 contained cells with the G_1_ phase being dominant in the cell cycle, but in the N-KYOU p25, the proportion of such cells dropped. In the G_1_ phase, cells choose their fate: to exit the cell cycle or to continue further rounds of division. During terminal differentiation into neurons or glia, progenitor cells take different fates depending on which differentiation signals they receive, with receptor signals in the early or late G_1_ phase being crucial for such cell differentiation (Pauklin and Vallier, 2013; Hardwick and Philpott, 2014). Thus, according to cell cycle duration theory, the length of the G_1_ phase determines whether the fate-determining signal will have enough time to produce an effect (Calegari and Huttner, 2003), lengthening the G_1_ phase leads to increased neuronal marker expression and to correct differentiation, whereas shortening G_1_ promotes proliferative divisions and the maintenance of precursor states (Lange et al., 2009). McConnell and Kaznowski (1991) demonstrated that immature cells can change their fate according to the microenvironment during transfer, but only if they are transplanted in their G_1_ or S phases, whereas cells transplanted in the M phase retain their early fates.

## Discussion

The Dual SMAD inhibition method allows the production of NSCs from PSCs. NSCs can be further directed to neuronal or glial differentiation. In general, it is known that such neural cultures differentiated from NSCs, even with directed differentiation, are heterogeneous. Moreover, the ratio of cell types in cultures derived from different NSC lines may also differ (Wu et al., 2007; Hu et al., 2010) and there is also a suggestion that the spectrum of cell types in neural cultures differentiated from such NSCs may vary depending on the duration of NSCs cultivation (Viero et al., 2014).

We decided to investigate the variability of neural cultures depending on the iPSC source NSC lineage as well as on the duration of NSC cultivation using an example with spontaneous differentiation of Dual SMAD-derived NSCs (by elimination of factors directing differentiation and factors supporting NSC proliferation). As expected, with the same protocol of NSCs production and spontaneous NSCs differentiation of the same duration of cultivation, different iPSC lines (KYOU, AFS17, DYP0730, DP) yield heterogeneous neural cultures, with varying degrees of heterogeneity. The source of these differences is likely to be the genetic and epigenetic properties of iPSC lines. It has been repeatedly noted that different PSCs lines can differ and, as a consequence, show a different spectrum of cell types during their differentiation. For example, it has been shown that different human iPS cell lines differentiate into NSCs with increased variability compared to different ESC lines (Hu et al., 2010; Kim et al., 2010; Bock et al., 2011). At the same time, such differences in the differentiation of different lenti- or retroviral transduction-derived iPSC lines did not depend on the possible residual expression of transgenes (Hu et al., 2010). It cannot be said that different ESC lines always show the same differentiation spectrum. For example, a comparison of the neural differentiation of two ESC lines showed a significant difference in the ratio of cell types in differentiated neural cultures (Wu et al., 2007). It is assumed that the lines are differentially programmed already at the PSC stage. This is expressed in the fact that although the cells of different PSC lines have the potential to differentiate into cells of the three germinal layers, but under the same conditions they can give preference to differentiation into a certain cell type, and it can be different for each line (Wu et al., 2007; Hu et al., 2010). Such pre-programming is probably related to epigenetic differences in cell lines, such as DNA methylation, histone modifications, and microRNA expression. The methylation status of a number of genes has been shown to differ between ESCs and iPSCs (Bock et al., 2011; Koyanagi-Aoi et al., 2013). This suggests the presence of epigenetic memory of somatic cells from which iPSCs were derived (Kim et al., 2010; Ohi et al., 2011). The same fact also explains the increased variability between iPSC lines compared to ESCs. In addition, the method of reprogramming also contributes to the potential differences. Reprogramming with pluripotency transcription factors can lead to genomic instability and mutagenesis, which contributes to the heterogeneity of the obtained iPSCs (Zheng et al., 2013; Ma et al., 2014). Thus, iPSC lines derived from different donors, or from different cell types, may show differences or limitations in differentiation spectra under the same conditions (Ohi et al., 2011; Kajiwara et al., 2012). Interestingly, for the most part, this is the case for PSCs obtained by reprogramming with transcription factors but not by core transfer (Kim et al., 2010).

Next, we investigated the change in the spectrum of spontaneous NSCs differentiation with increasing duration of cultivation (up to 25 and 30 passages) on two iPSC-derived NSCs (NSC-KYOU and NSC-AFS17). Our results showed that long-term cultivation of NSCs leads to changes in the transcriptional profile in spontaneously differentiated from them neuronal cultures. And the dynamics of these changes are not the same in neural cultures of two different lines. In particular, it was observed that with increasing duration of NSCs cultivation in N-KYOU a decline in glial markers (GFAP, CD44, s100b, etc.) occurs, while in N-AFS17, their expression profile increases. Nevertheless, both lines can be united by similar dynamics of some processes. The combined analysis of two cell lines suggests involvement of depletion of WNT and BMP signaling pathways in apparent difference between early and late passaged cells. The WNT pathways are essential in homeostasis formation between neuronal and glial cells (Lie et al., 2005; Moreno-Estellés et al., 2012). In the normal developing of a brain WNT signals via induction of BMP signaling gradually fade away which results in neurogenesis being replaced by gliogenesis which in turn becomes more skewed toward oligodendrocytes vs. astrocytes (Kasai et al., 2005; Gámez et al., 2013). In our artificial system we observe similar pattern, where some of WNT/BMP genes became less expressed with passages (*BMP5, RNF43* and others), WNT inhibitor *SHISA3* is upregulated in the stem cell clusters, astroglial cells became depleted, and some markers of oligodendrocytes became more expressed (most notably, *PLP1*).

Finally, using scRNA-seq analysis, we confirmed that with increasing length of NSC cultivation in neural cultures, changes occur not just at the level of transcriptional profiles, but at the level of cell populations. The major observed effect in our analysis of cellular composition of N-KYOU with scRNA-seq data was the dramatic depletion seen in passages of astroglial clusters 0 and 2. An interesting and discussable result is that despite the dramatic decrease in glial differentiation clusters in N-KYOU, we did not observe the same effect for neuronal differentiation clusters. It is clear that NSCs are capable of giving rise to both neurons and glial cells, and that the direction of cell fate can be manipulated by changing the cellular environment to direct differentiation into certain phenotypes. However, in our experiments, we intentionally did not add specific chemicals (such as FGF10, retinoic acid, etc.) in order to observe the spectrum of spontaneous differentiation. And under these conditions, despite 25 passages, we saw the correct passage of the entire differentiation pathway of N-KYOU from cluster 4, through clusters 7, 11, 3, 17, 1 to the states of clusters 5, 16, 14 which apparently characterize the most mature neurons of different types. We can assume that in this case the program for triggering terminal differentiation into precursor neurons does not depend so much on proliferation as on the program for terminal glial differentiation. At the same time, according to the data obtained from bulk transcriptomes, we see a significant and reliable decrease in the expression of mature neuronal marker genes (*GAP43*, *RBFOX3* (NeuN), *SYN*, *TH*, *SLC6A1*, *GAD2*, etc.). Perhaps this also indicates a decrease in the differentiation potential of NSCs toward mature neurons as well. Certainly, however, this question should be studied in more detail. The general theory of neurogenesis suggests that as the human nervous system develops during embryogenesis the wave of neuron formation should strongly outpace waves of glia formation (astrocytes, followed by oligodendrocytes) (Miller and Gauthier, 2007), but in N-KYOU cultures we observe a different picture–constant neuron generation with attenuation of glia generation. Our data agrees well with the published observation that astrocytes are correctly differentiated from iPSC-derived NSCs in culture only up to the 14th passages (Tcw et al., 2017). Of course, long-term cultivation of neural stem cells, under the conditions of our experiment, does not simulate normal human neurogenesis, not least for the reason that the development of the human fetal nervous system takes many months, including the postnatal period. On the other hand, histological data confirm the ratio of glia to neurons as approximately to 1:1 in the entire human brain. Our scRNAseq results showed that the ratio in N-KYOU p25 is more similar to the natural ratio than that in p5 (1.1 versus 1.8). To more directly correlate the сell model with natural neurogenesis, better three-dimensional cell maturation conditions, such as neurospheres and neuro-organoids, are probably needed. Nevertheless, there are experiments showing that increasing the maturation time (differentiation) of PSC-derived NSCs as neurospheres from 8 to 15 weeks increases the proportion of astrocytes (Paavilainen et al., 2018).

Our study has several important limitations. In spite of the fact that we observed the differentiation spectrum shift by several independent methods of analysis in a number of independent biological replicates, methodologically, our experiment was limited to a specific protocol of NSC differentiation from a particular iPSC lines. It is quite possible that the application of other protocols on other similar cell lines could affect the result, because in this case we are working with systems with a large number of variables (genetic and epigenetic background, the properties of different batches of the culture media and supplements used, specifics of culturing by laboratory staff, etc.). Nevertheless, even on such a limited set of cell lines, we demonstrated significant differences in the spectrum of differentiated cells both derived from different iPSC lines and over the course of long-term cultivation. Investigating the mechanisms of glial differentiation in iPSC-derived NSCs and identifying reasons for suppressed terminal differentiation during long-term cultivation are valuable areas for future research. Understanding the detailed mechanism of glial differentiation of NSCs could also help the development of cell technologies in regenerative medicine.

## Conclusion

We investigated how the spontaneous differentiation potential of human neural stem cell cultures derived from different iPSC lines changes over increasing passages *in vitro*. Using scRNA-seq analysis, we confirmed the heterogeneous composition of such neural cultures and found a significant shift in the spectrum of differentiated cells, with one culture showing a reduced ability to differentiate into glial cells. Our findings further contribute to the understanding of the biology of human neural stem cells and how they can be cultured under *in vitro* conditions, which could affect the strategy of using these cells for transplantation in regenerative medicine.

## Data availability statement

The datasets presented in this study can be found in online repositories. The names of the repository/repositories and accession number(s) can be found at: https://www.ncbi.nlm.nih.gov/bioproject/846149.

## Author contributions

ED, VG, and IZ designed and supervised the study. AG, AA, and EM developed the methodology and performed experiments. NK performed bioinformatic analysis of bulk RNA-seq and LDSC. OB, OK, and AL performed bioinformatic analysis of scRNA-seq. BS and AZ performed quantitative analysis of cell samples after immunocytochemical staining. ED, AG, AA, OB, NK, OK, and AL interpreted the data and wrote the manuscript. All authors have read and approved the manuscript.

## Funding

This research was funded by Grant No. 075–15–2019-1789 from the Ministry of Science and Higher Education of the Russian Federation, allocated to the Center for Precision Genome Editing and Genetic Technologies for Biomedicine. RNA-seq and LDSC analysis was supported by Russian Science Foundation Grant No. 21–15-00124, https://rscf.ru/project/21-15-00124. The work of AA was supported by the Systems Biology Fellowship provided by Skoltech.

## Conflict of interest

LO was employed by AcademGene LLC.

The remaining authors declare that the research was conducted in the absence of any commercial or financial relationships that could be construed as a potential conflict of interest.

## Publisher’s note

All claims expressed in this article are solely those of the authors and do not necessarily represent those of their affiliated organizations, or those of the publisher, the editors and the reviewers. Any product that may be evaluated in this article, or claim that may be made by its manufacturer, is not guaranteed or endorsed by the publisher.

## Footnotes

^1^ https://coletrapnelllab.github.io/monocle3/

^2^ https://www.gseamsigdb.org/gsea/downloads.jsp

^3^ https://github.com/bulik/ldsc/wiki

## Abbreviations

NSC: neural stem cell
iPSC: induced pluripotent stem cell
DE: differentially expressed
FC: fold change
TPM: transcripts per million
GSEA: Gene Set Enrichment Analysis
LDSC: LD Score Regression
BH-adjusted *p*-values: Benjamini-Hochberg adjusted *p*-values
