# Supplementary material for: Different iPSC-derived neural stem cells shows various spectrums of spontaneous differentiation during long term cultivation
Source: Front Mol Neurosci. 2023 May 2;16:1037902. doi: 10.3389/fnmol.2023.1037902 (PMC10186475; doi:10.3389/fnmol.2023.1037902)
Supplement: Supplementary file 2 [file Data_Sheet_1.docx]

SUPPLEMENTED MATERIALS

(Manuscript “Different IPSC-derived neural stem cells shows various spectrums of spontaneous differentiation during long term cultivation.” Galiakberova et al. 2023)


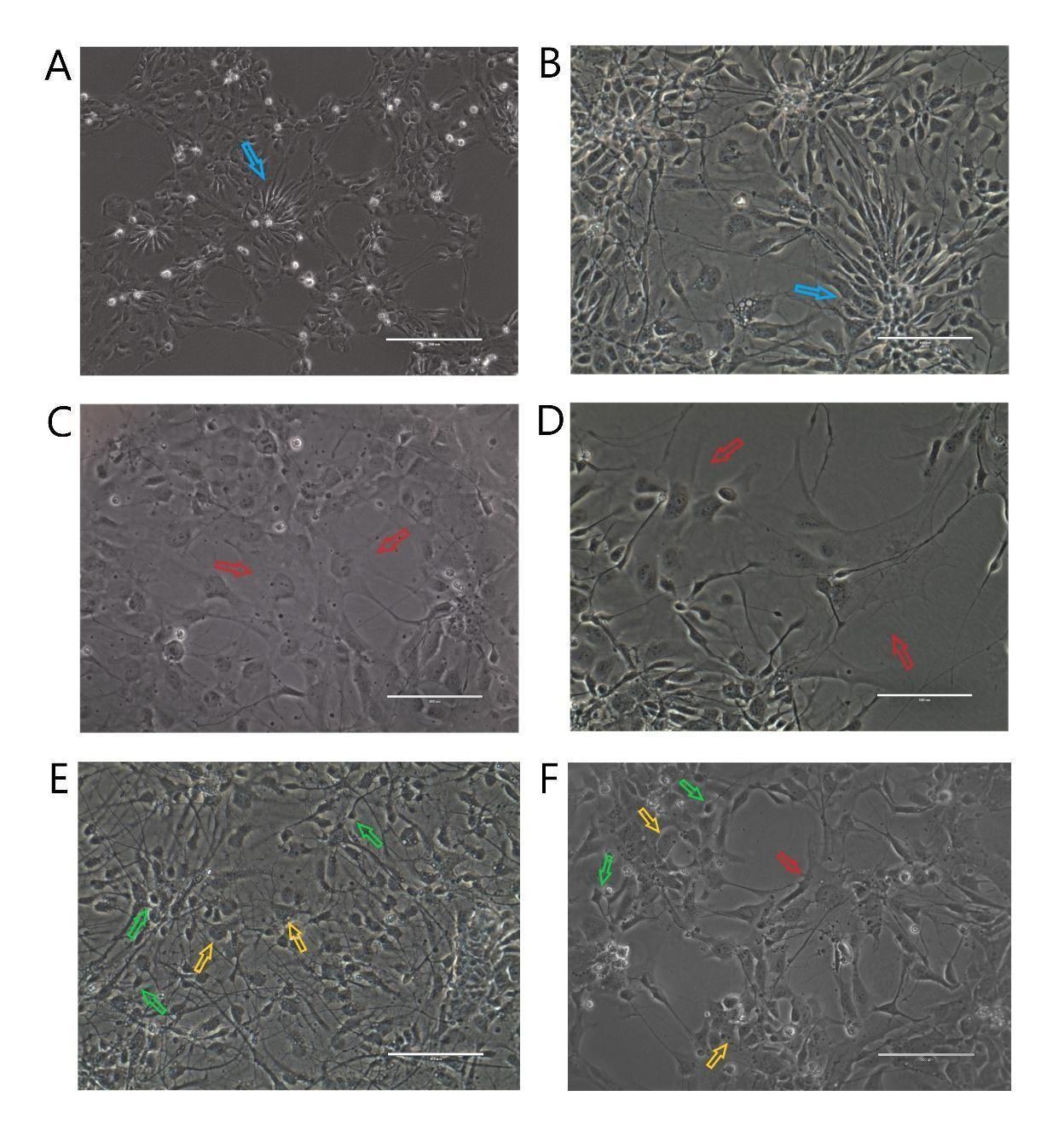


**Suppl.Fig. 1** Heterogeneity of the NSC-KYOU and neural culture from NSC-KYOU.

**A** - Neural rosettes in NSCs, are indicated by blue arrows ; **B** - Neural rosettes in neural culture are indicated by blue arrows,; **C** - Large cells with irregular flattened shape in NSC culture, are indicated by red arrows; **D -** Large cells with irregular flattened shape in neural culture, are indicated by red arrows; **E** - Neural culture at day 14 of spontaneous differentiation at passage 7, large non-neural cells — red arrows, NSC-like cells — yellow arrows, neuron-like cells — green arrows; **F** - Neural culture at day 14 of spontaneous differentiation at passage 25, large non-neural cells – red arrows, NSC-like cells – yellow arrows, neuron-like cells – green arrows; Phase contrast. Scale bar 200 μm (A), 100 μm (B-F)

A B


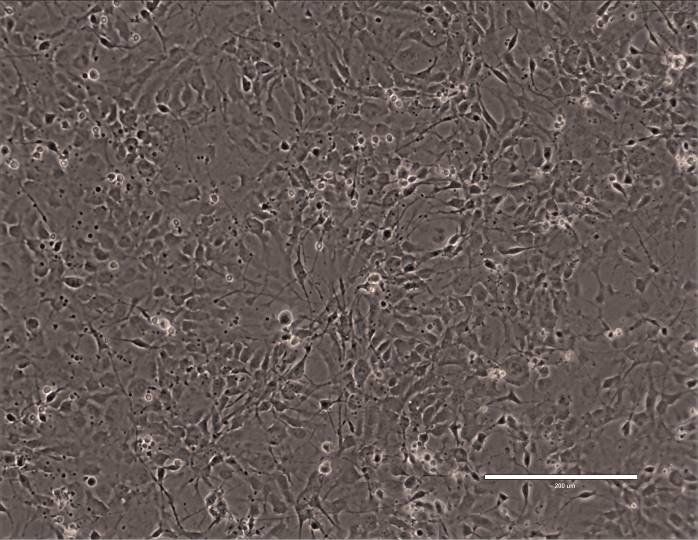

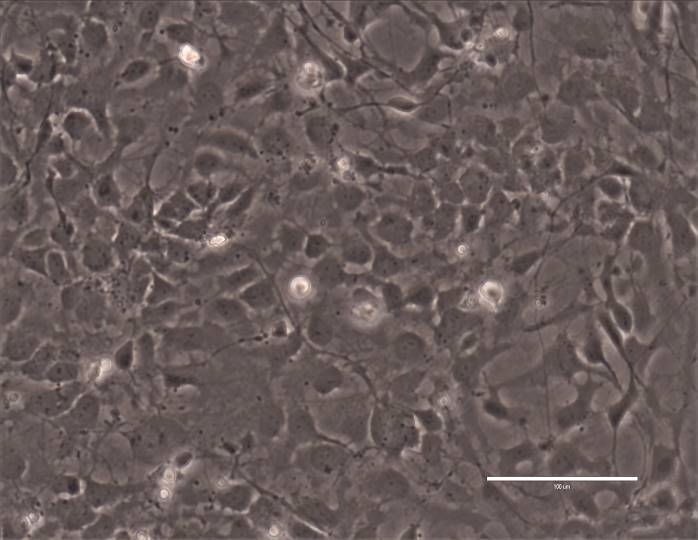


C D


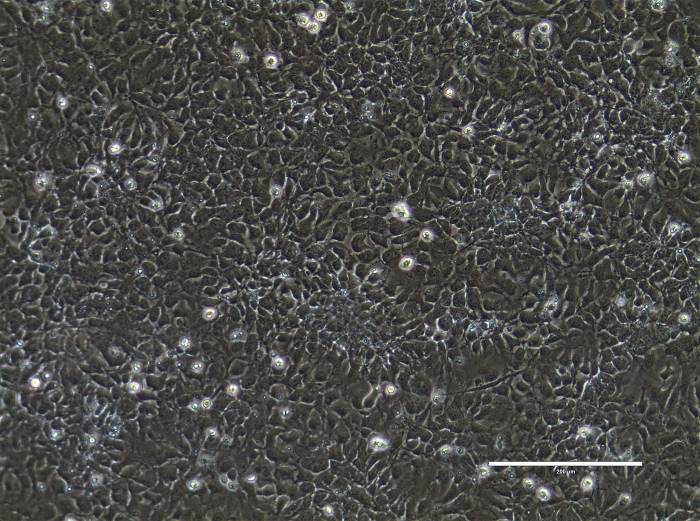

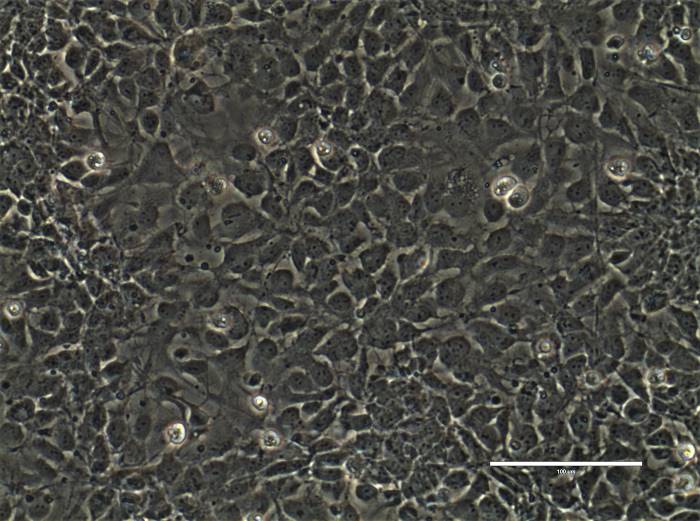


E F


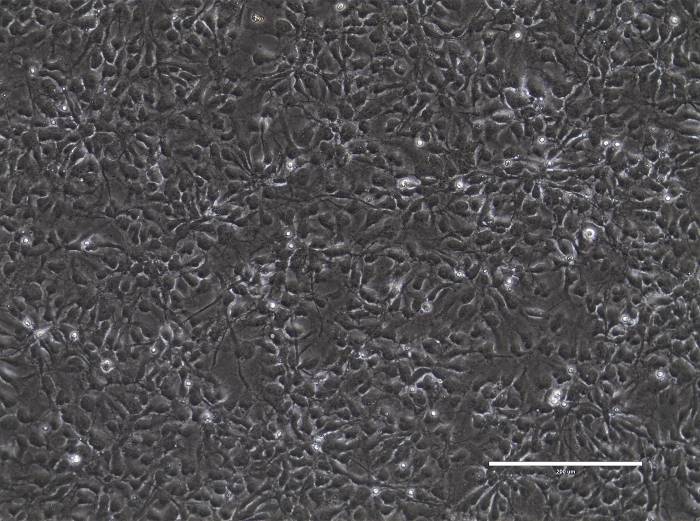

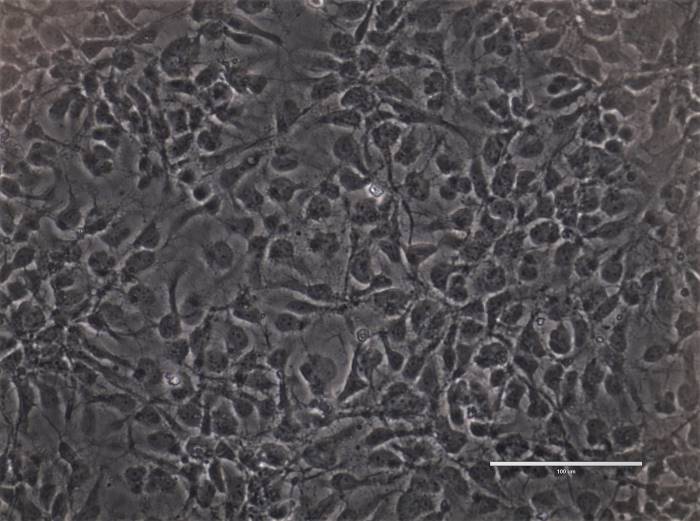


G H


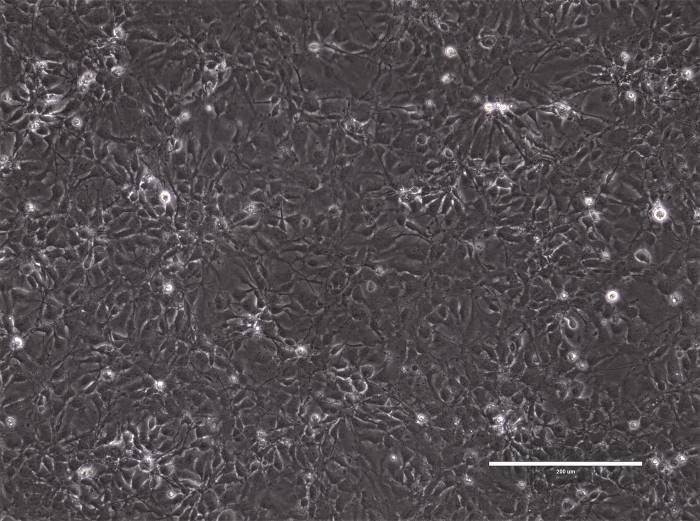

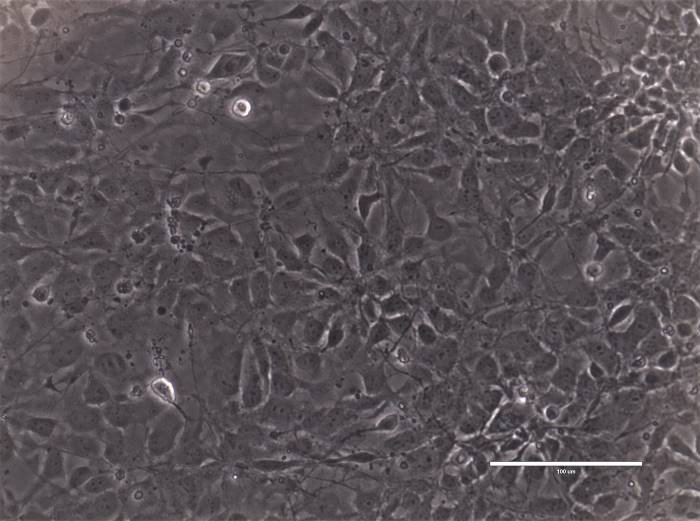


I J


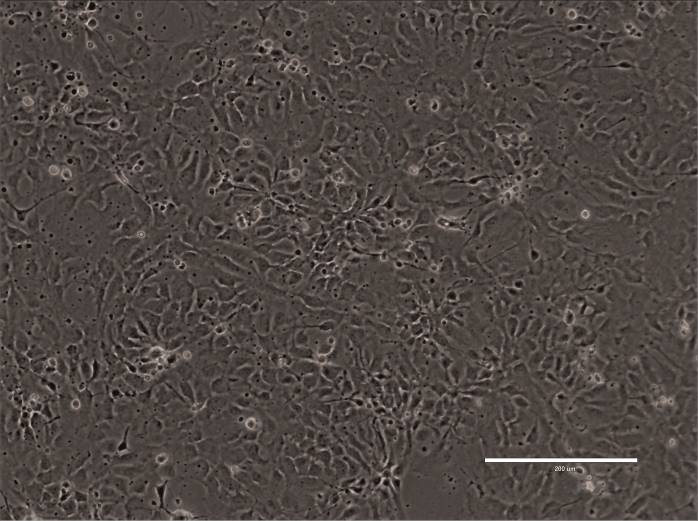

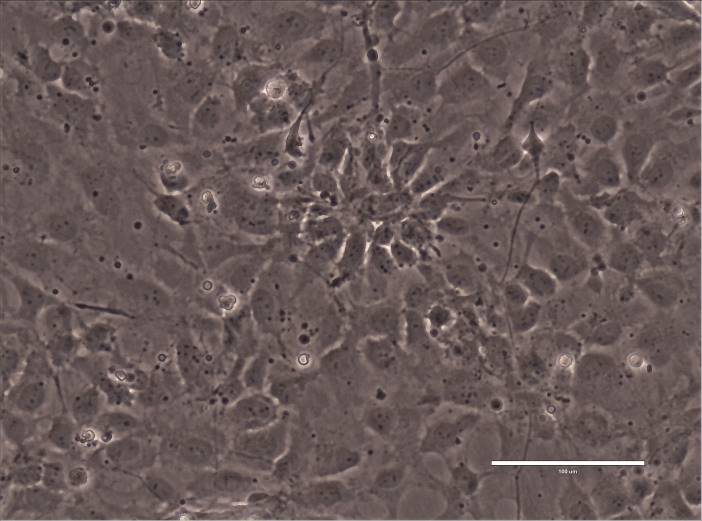


K


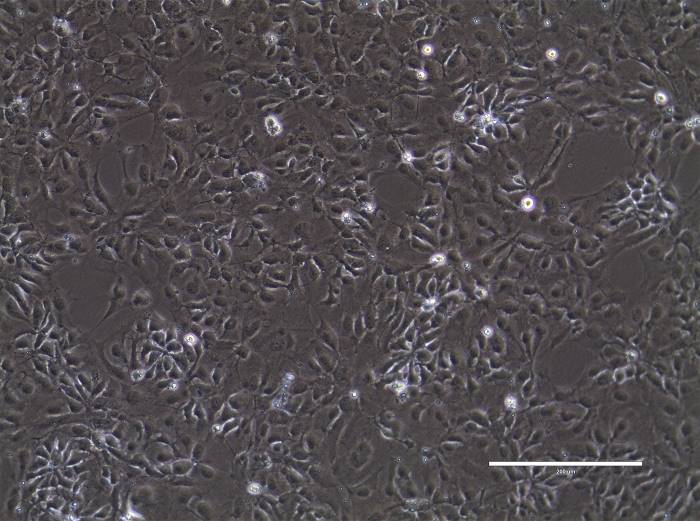


**Suppl.Fig. 2** Morphology of NSC-KYOU cells in phase contrast during long-term cultivation. **A**, **B** – passage 5; **C**, **D** – passage 10; **E**, **F** – passage 15; **G**, **H** – passage 20; **I**, **J** – passage 25; **K** – passage 30. Phase contrast. Scale bar 200 μm (A, C, E, G, I, K), 100 μm (B, D, F, H, J)

A B


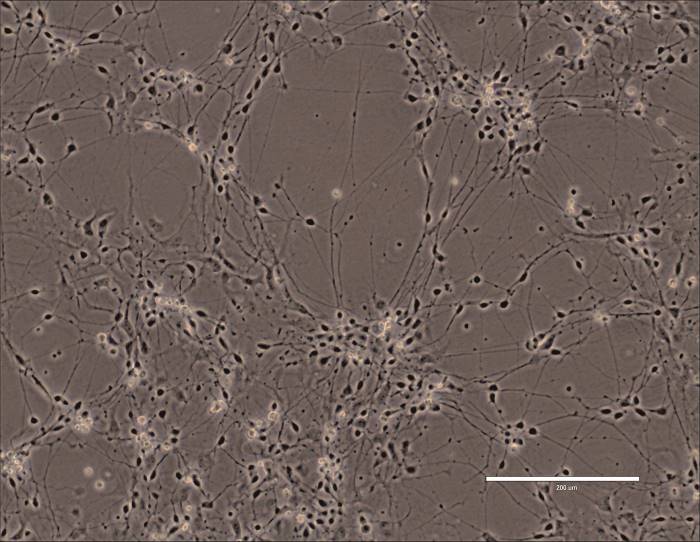

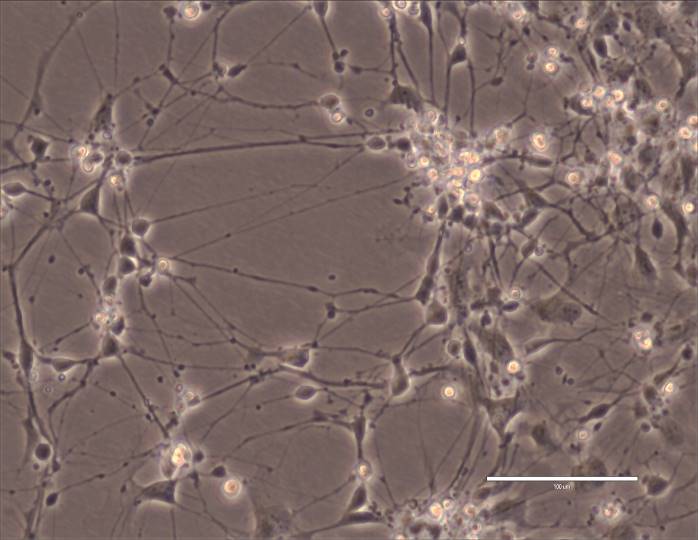


C
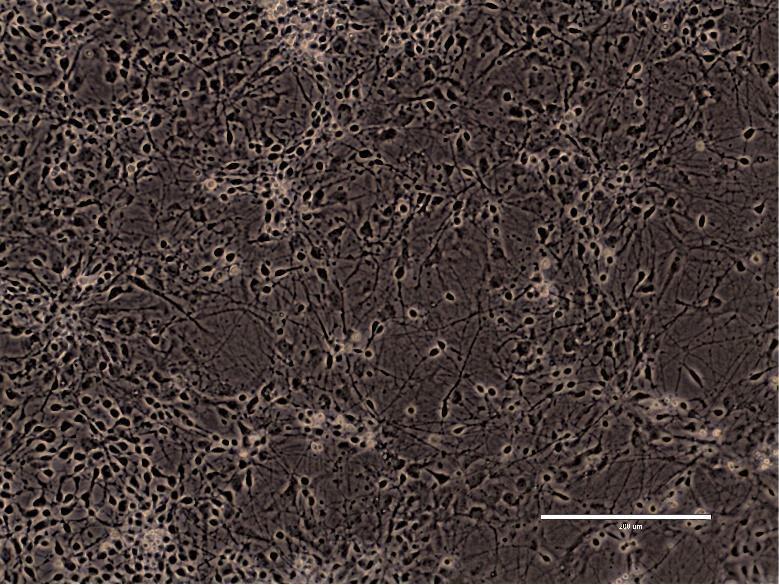
D
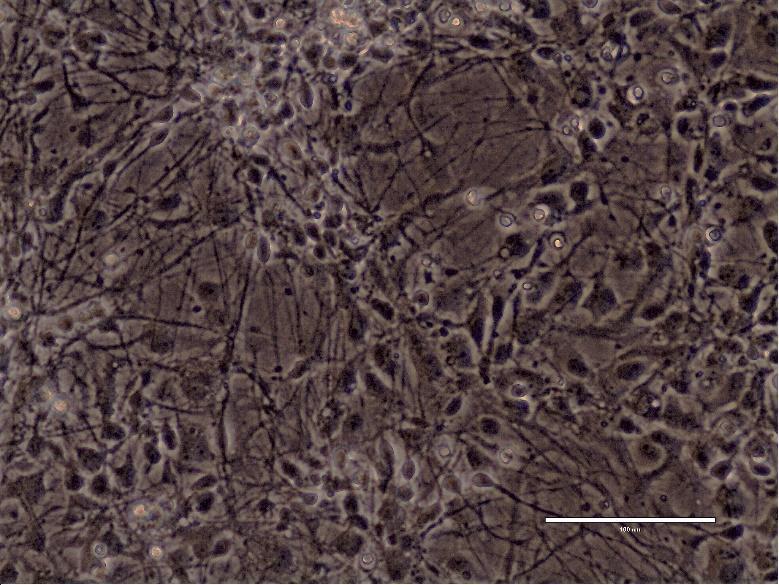


E F


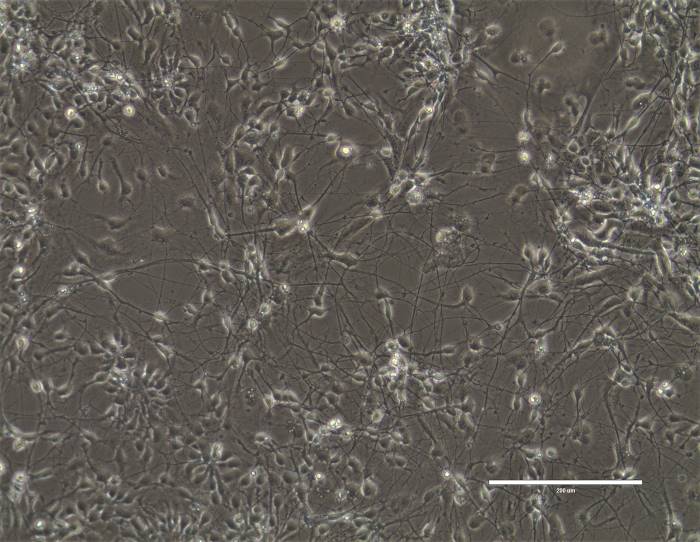

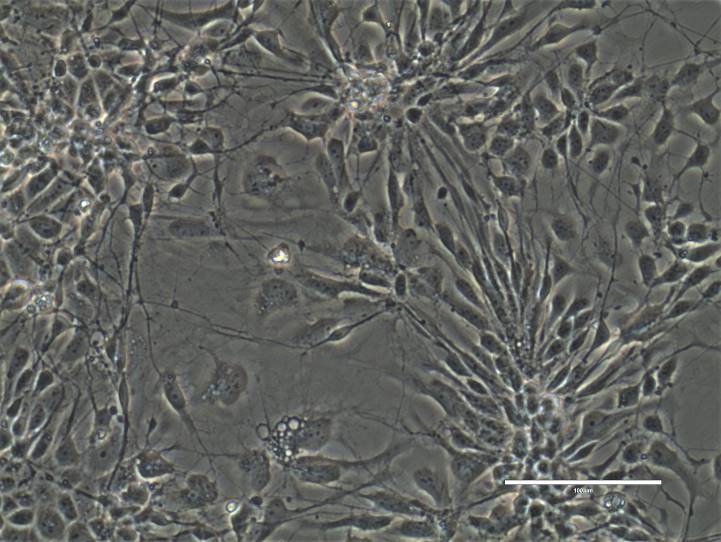


G H
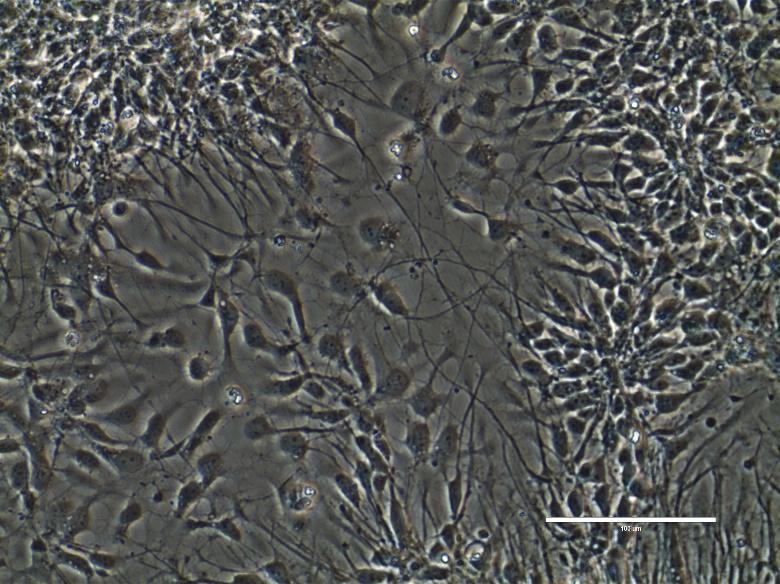


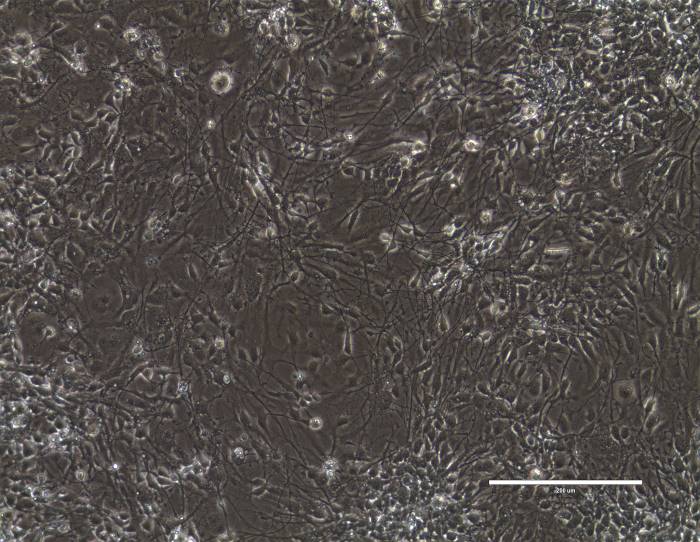


I
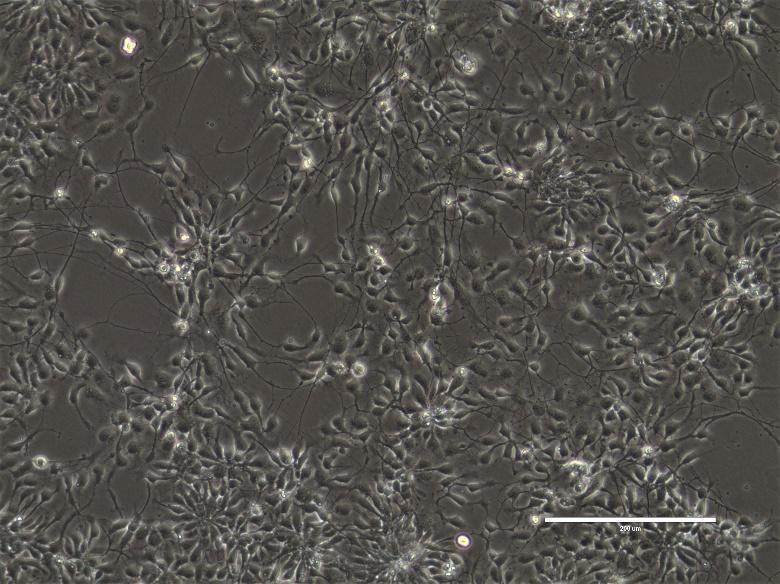
 J


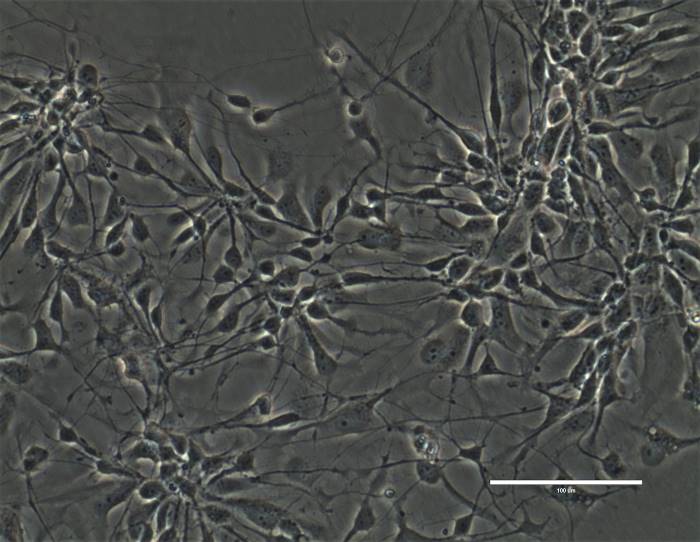


K
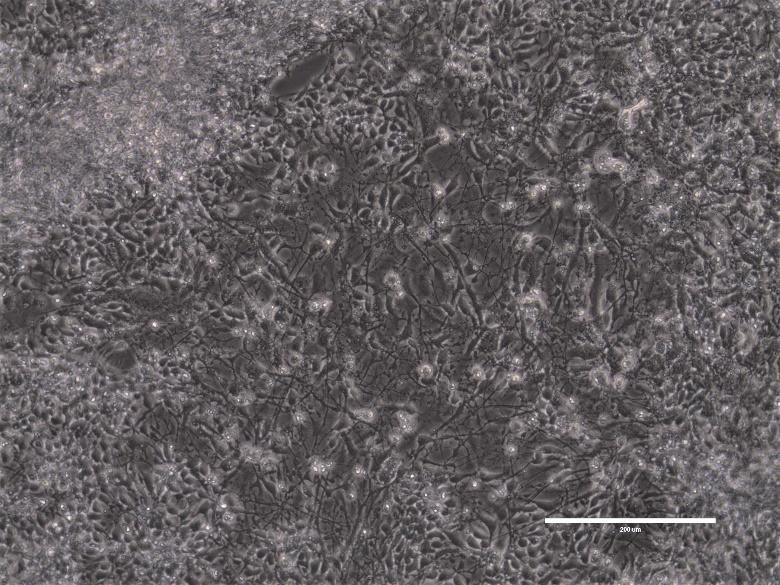
L
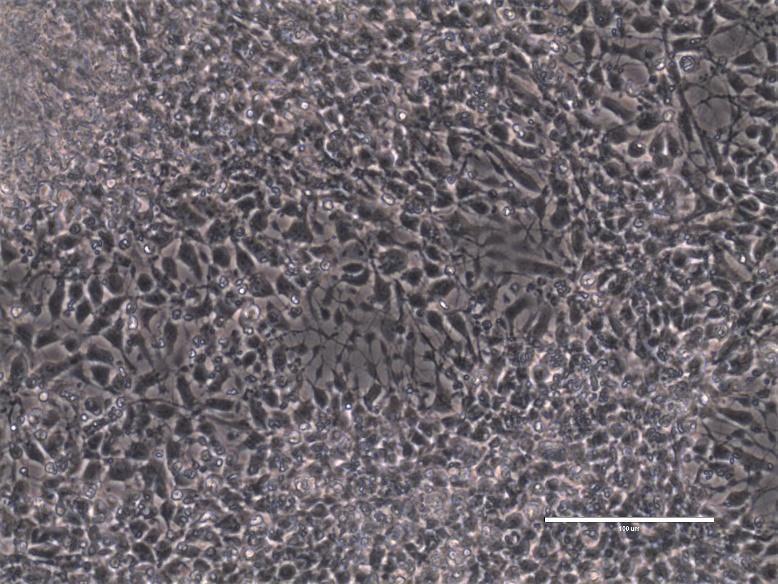


M N


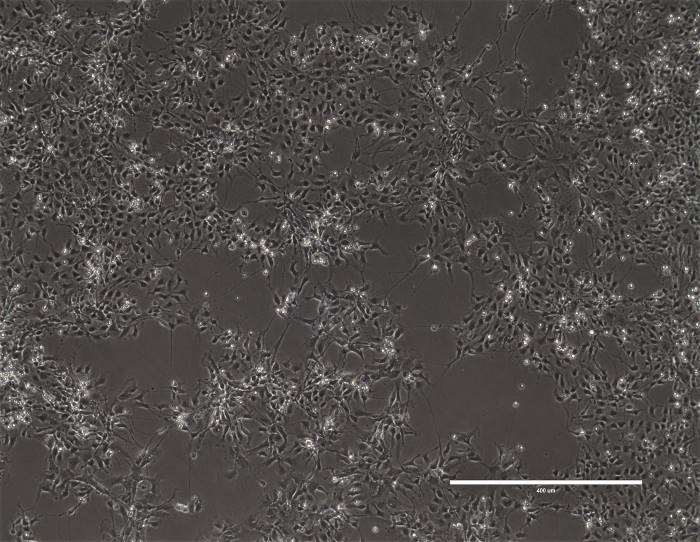

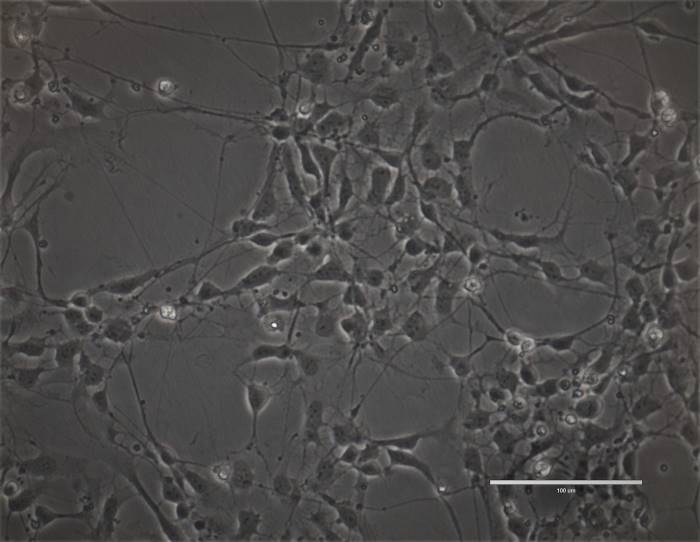


O
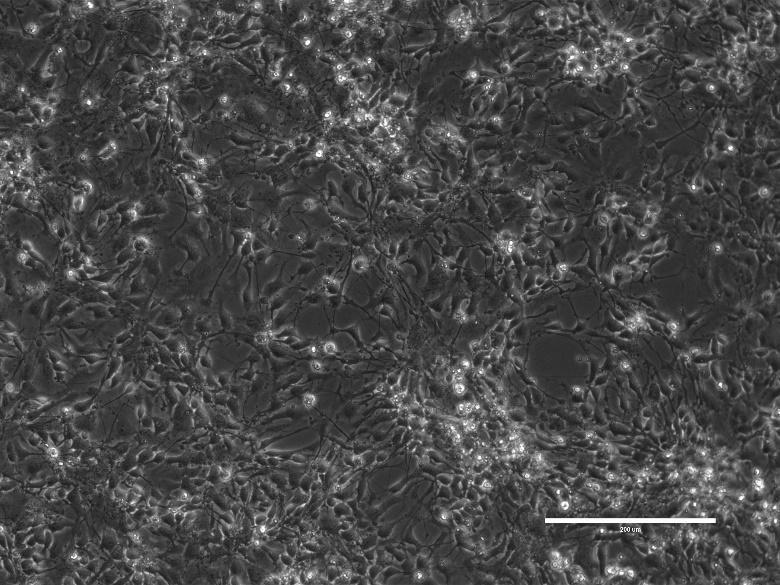
P


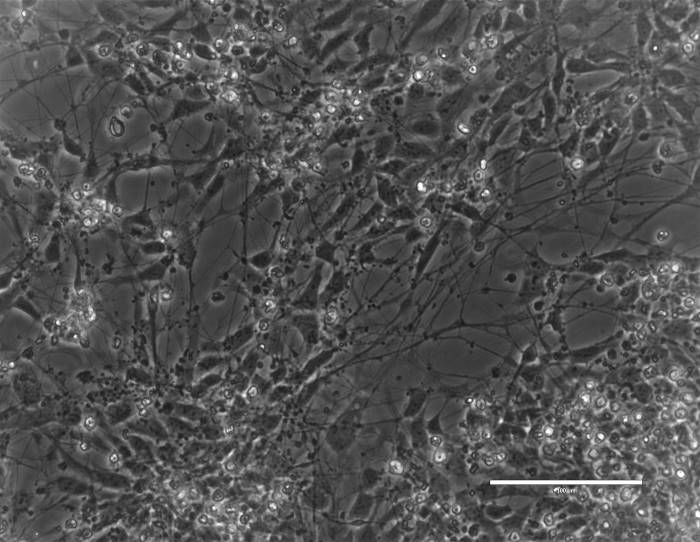


Q
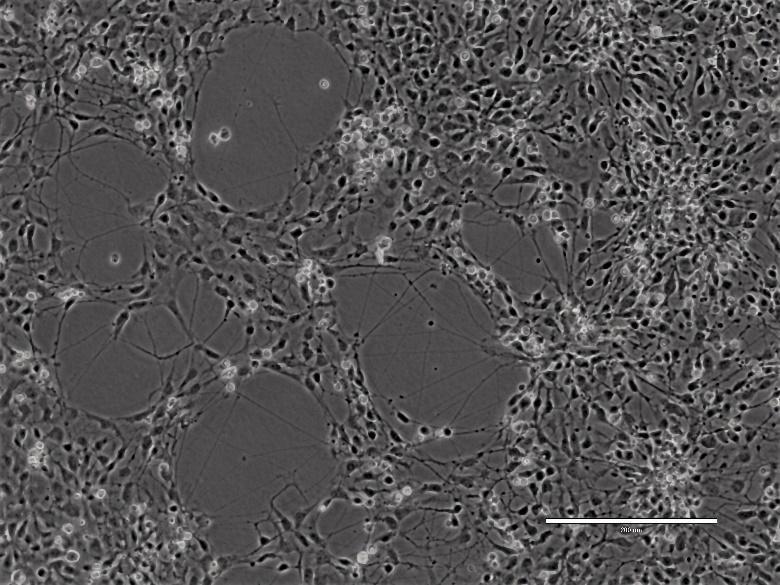
R
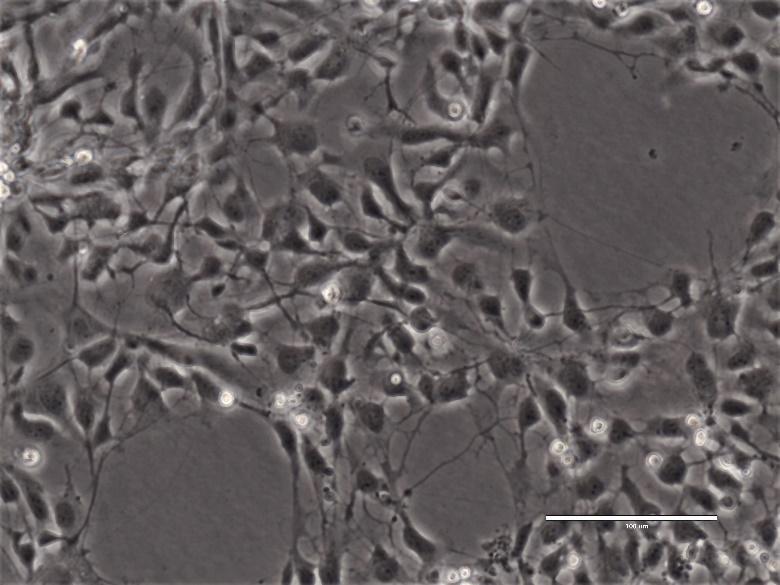


S
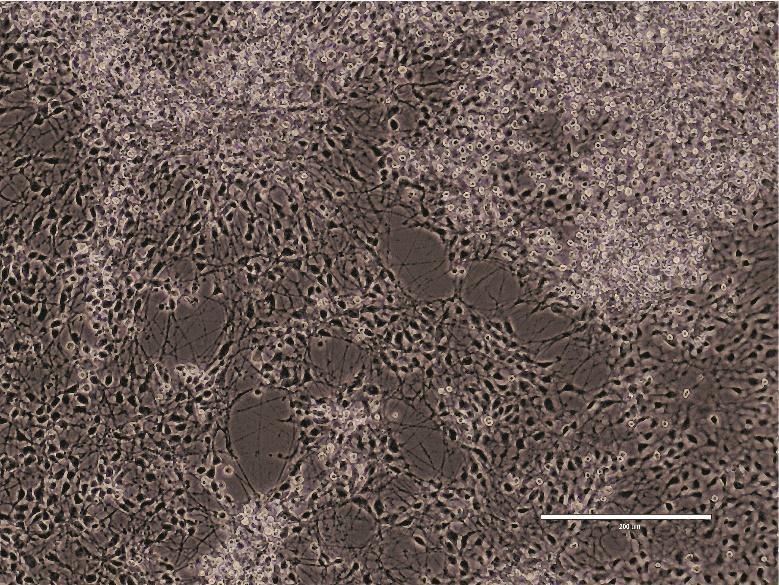
T
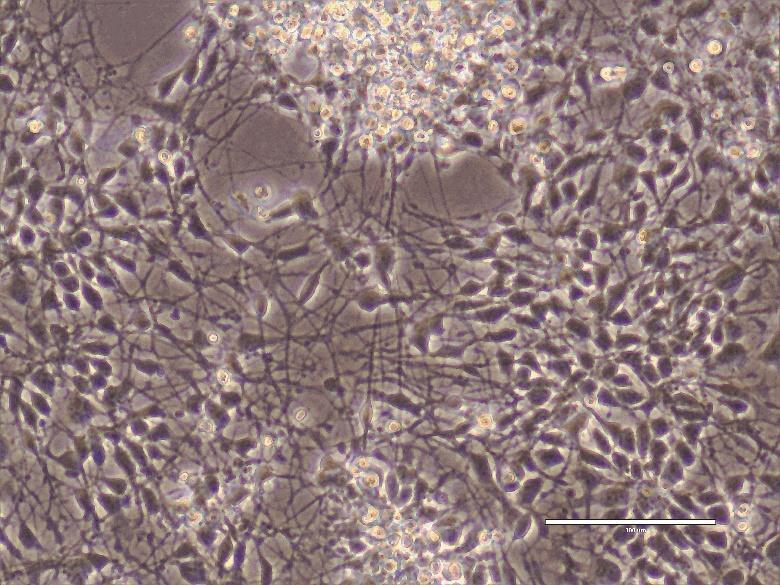


U
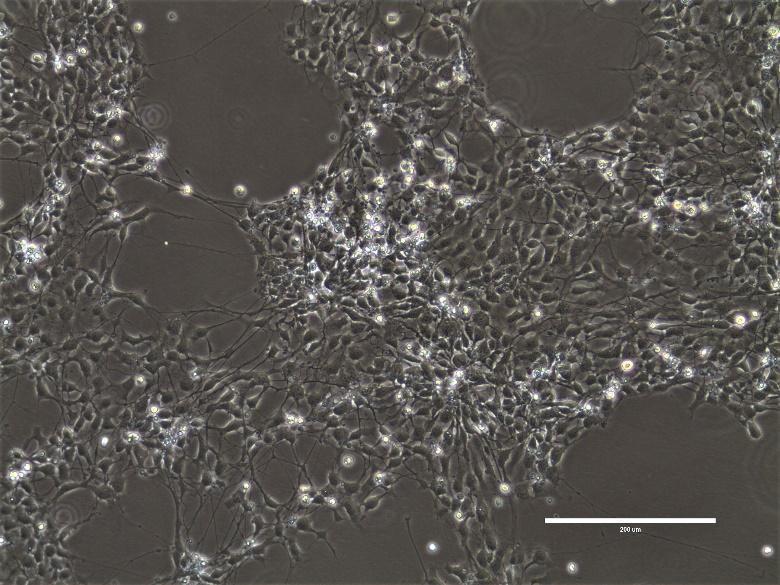
 V
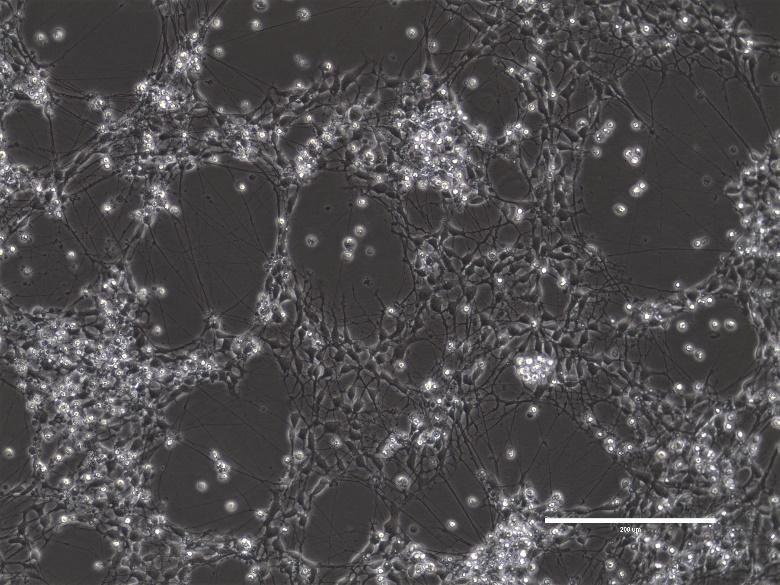


**Suppl.Fig. 3.** Morphology of neural cultures derived from NSCs of different passages at 7 (**A, B, E, F, I, J, M, N, Q, R, U**) and 14(**C, D, G, H, K, L, O, P, S, T, V**) days of spontaneous differentiation. **A**-**D** – passage 5; **E**-**H** – passage 10; **I**-**L** – passage 15; **M-P** – passage 20; **Q-T** – passage 25; **U-V** passage 30.

Phase contrast. Scale bar 200 μm (A, C, E, G, I, K, O, Q, S, U, V), 100 μm (B, D, F, H, J, L, N, P, R, T), 400 μm (M)

A B


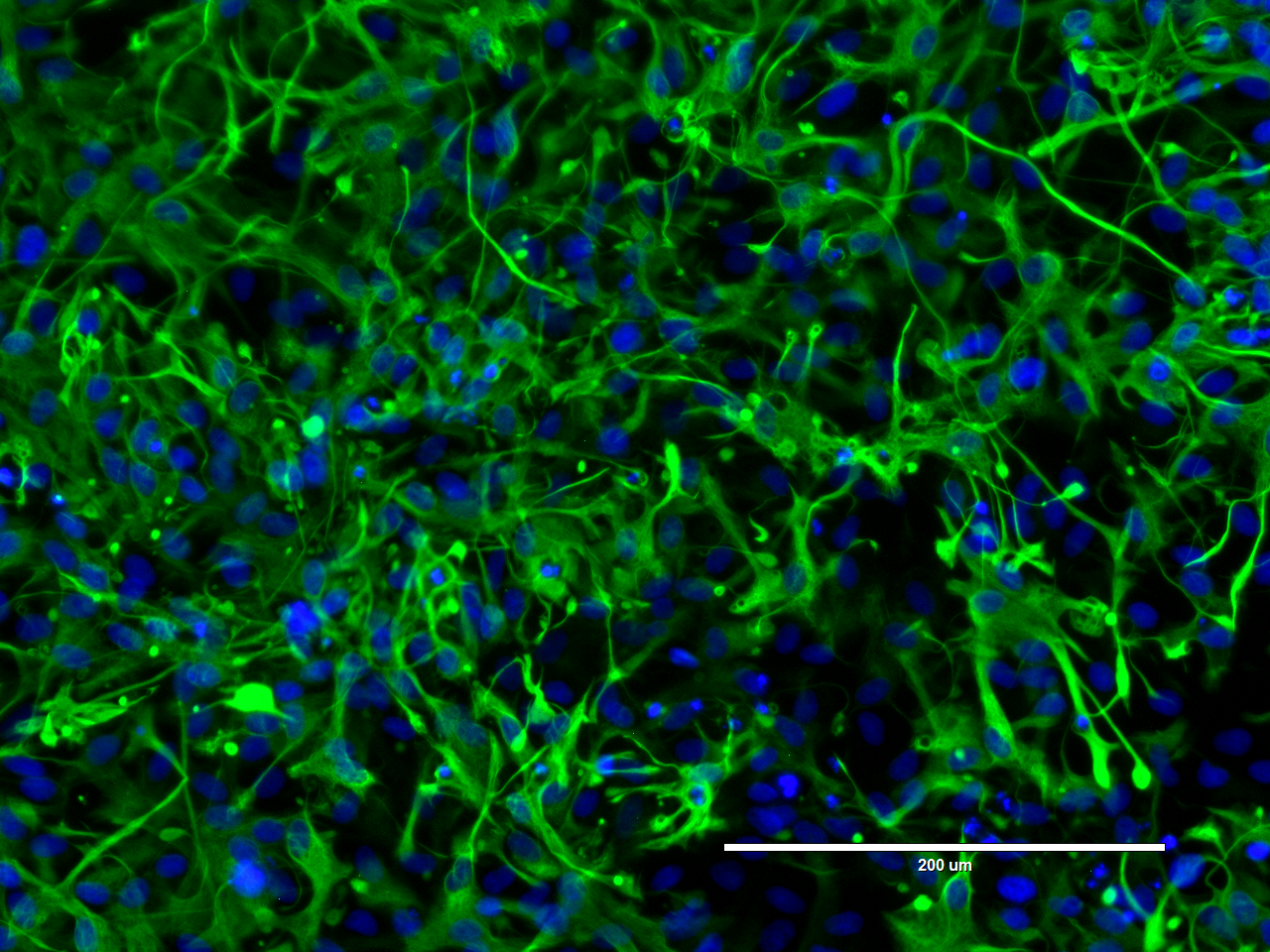

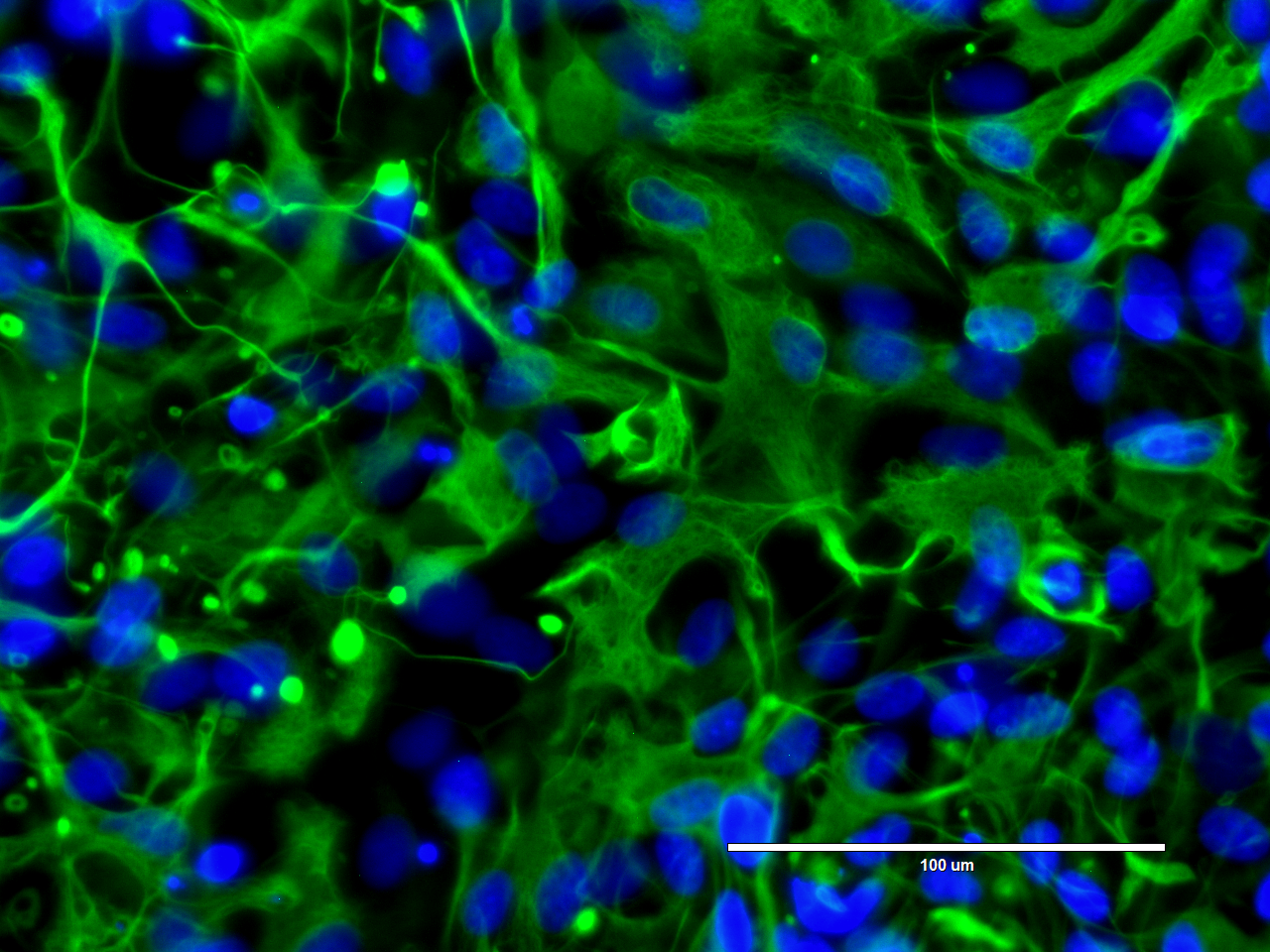


C D


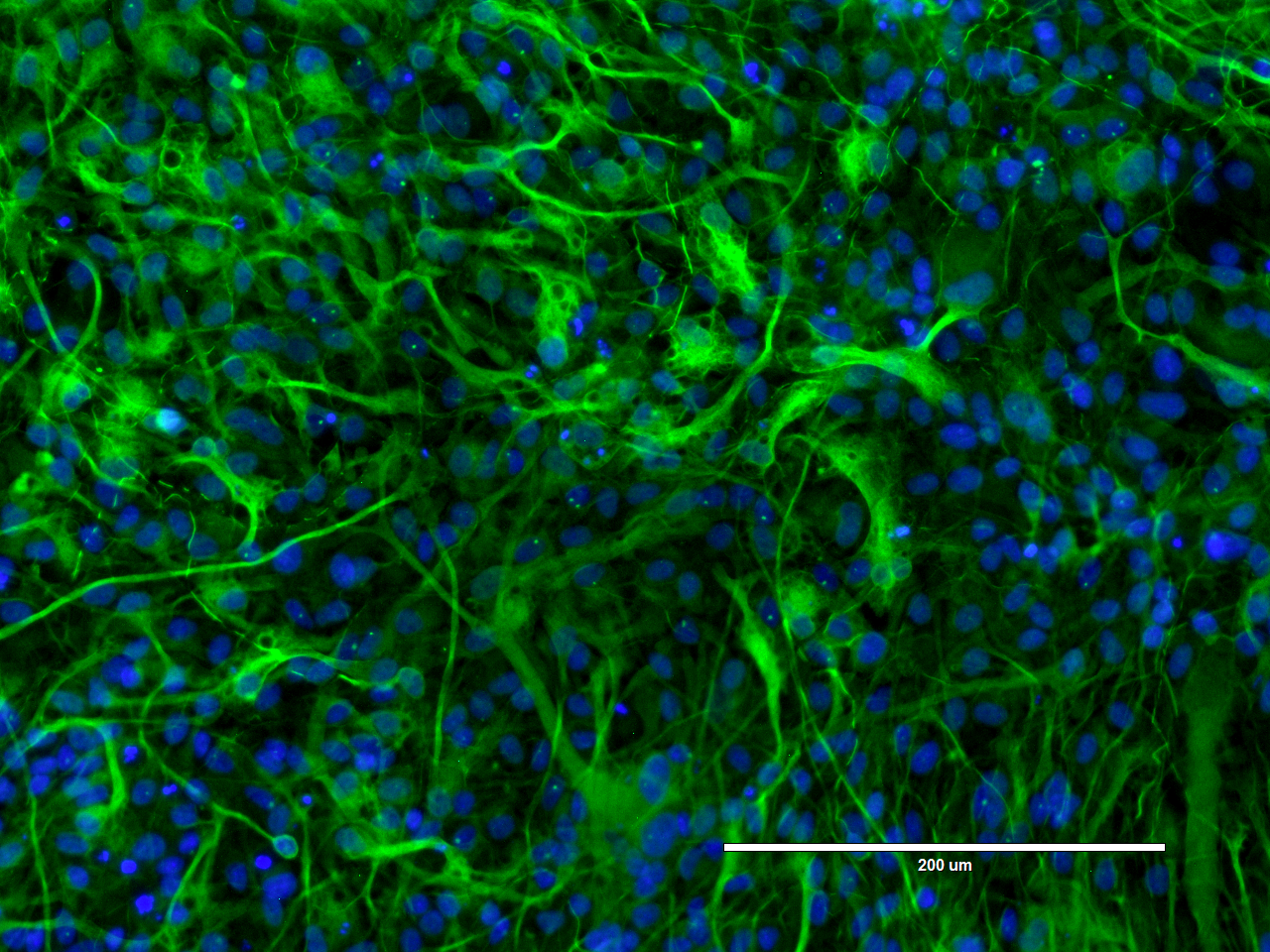

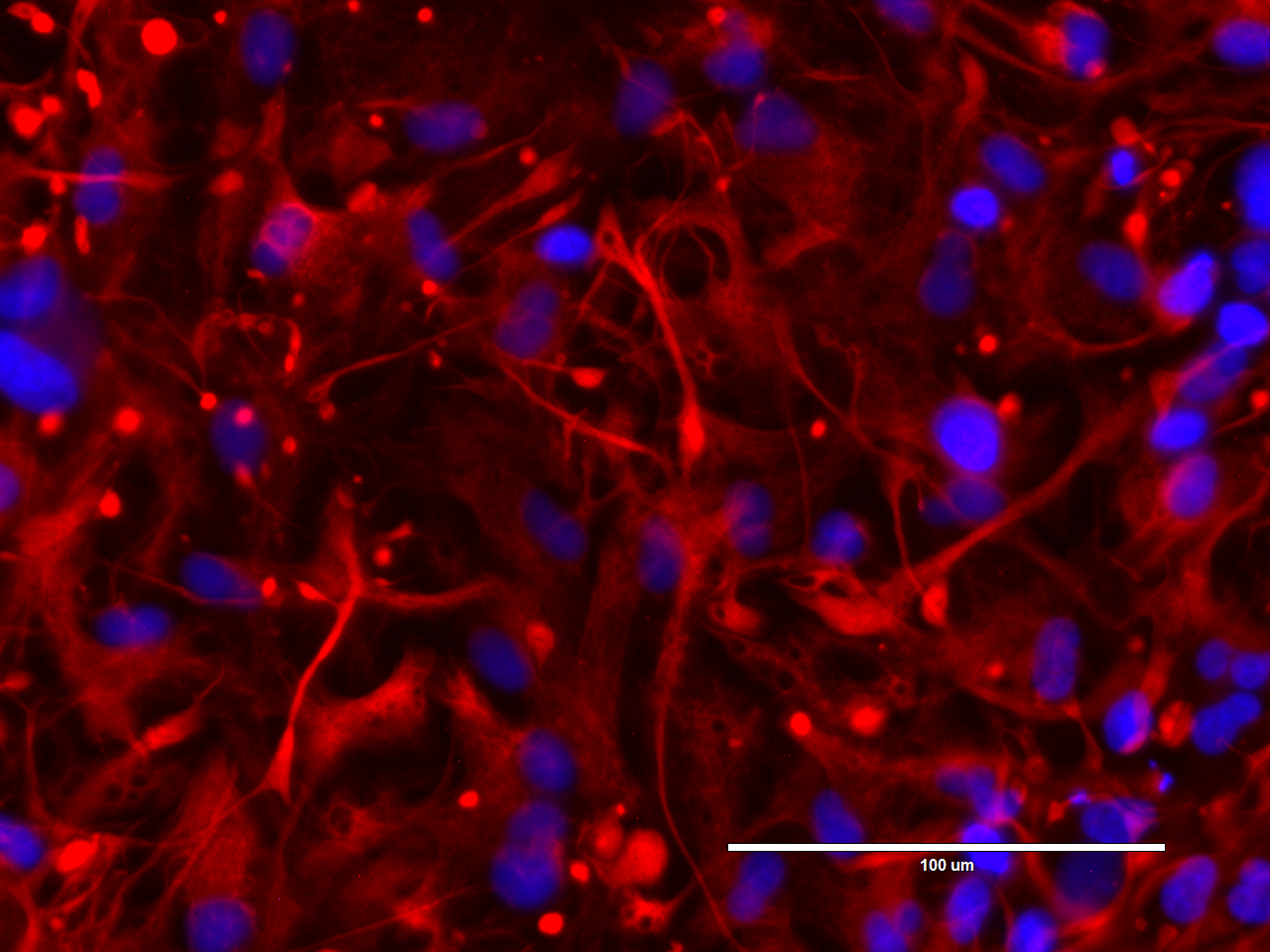


**Suppl. Fig. 4.** Morphology of DYP0730 neural culture cells stained for: **A, B** - GFAP, **C** - b-III-tubulin, **D** – Nestin. Fluorescence microscopy. Scale bar 200 μm (A, C), 100 μm (B, D).

***A***


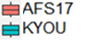

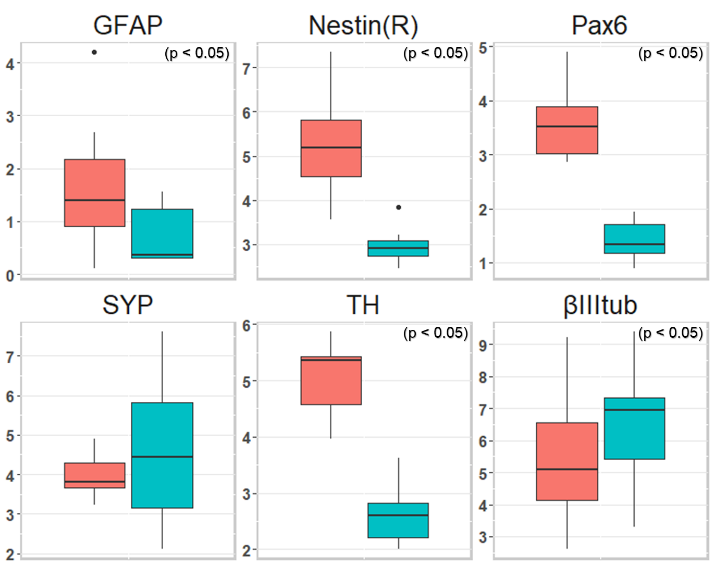


***B***


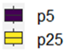

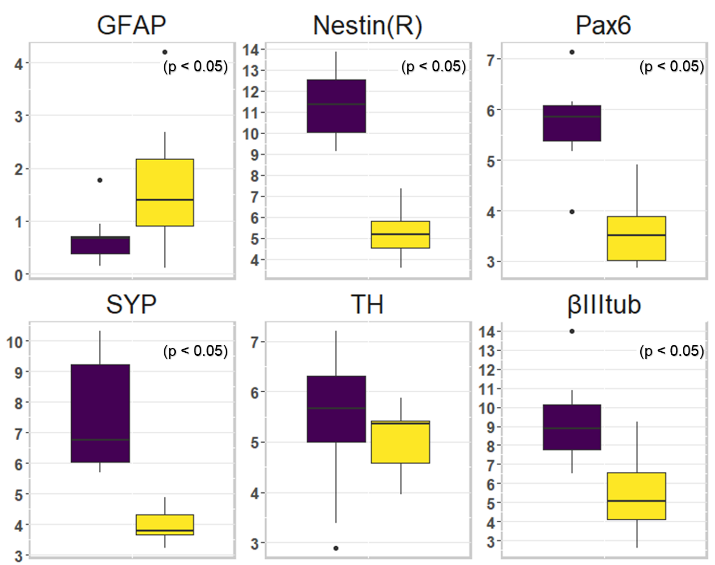

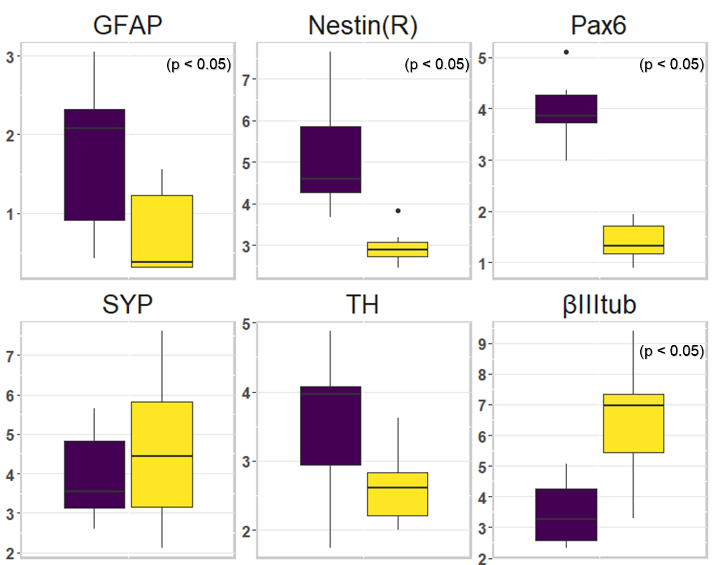


***D***

***C***


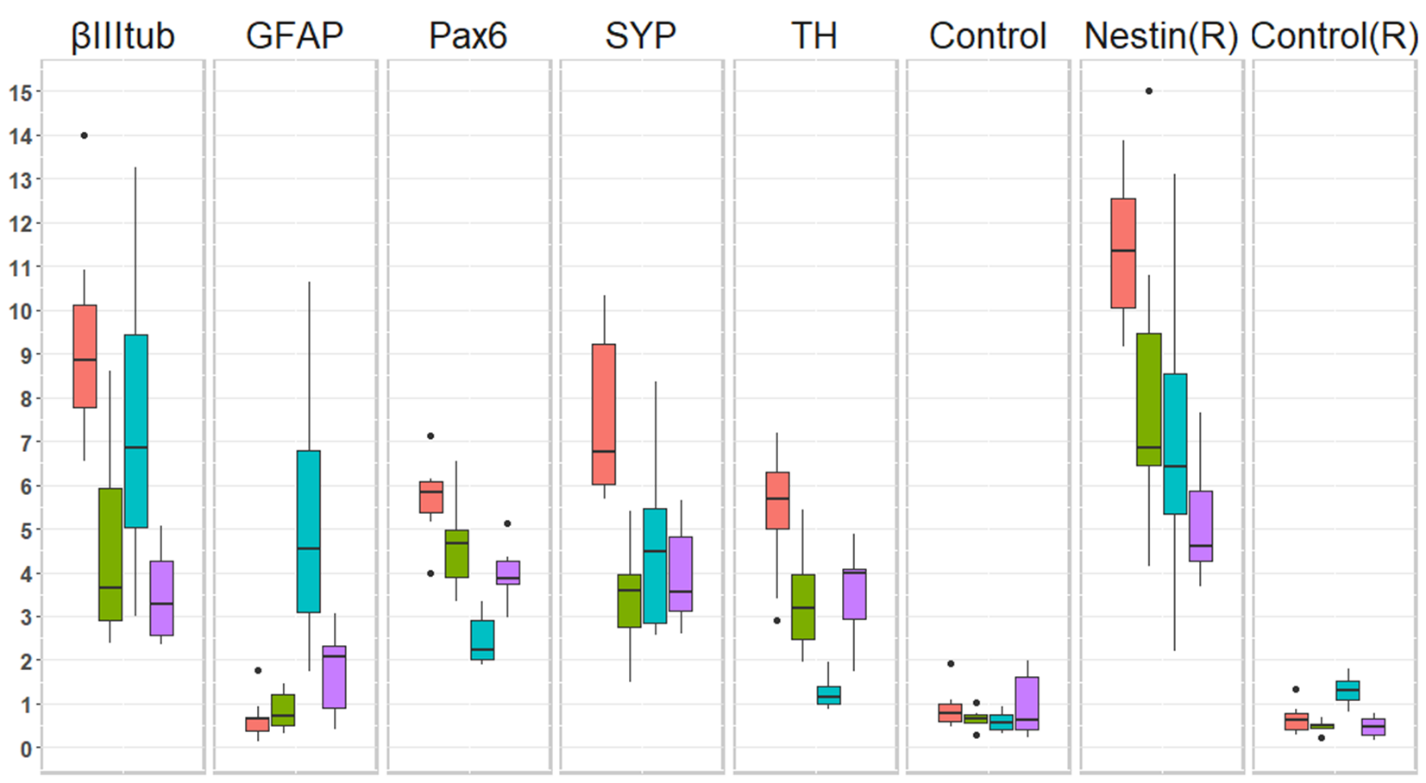

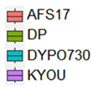


***E***

**Notes:** Groups — comparable cell lines / passages;
Datasets — size of groups, includes six antigens;
p-value — Mann-Whitney test result, corrected for the false discovery rate for multiple testing.


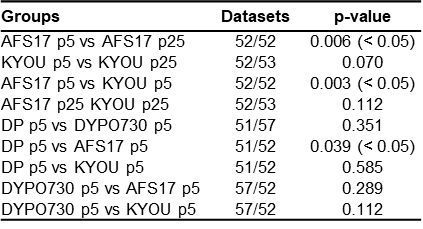


**Suppl. Fig.** **5**. Quantitative analysis of the IHC staining results. Groups - comparable cell lines or passages.
All markers stained with a second antibody with green fluorescence, except nestin, nestin stained with a second antibody with red fluorescence (R). The number of processed pictures used for statistical analysis is shown in Table S3.

(**A)** – p-values of two-sided Mann-Whitney pairwise comparisons with corrections for multiple testing.
**(B-E)** – Boxplots of the ratios grouped by the markers and the cell lines:

(**B**) Group of N-KYOU and N-AFS17 of 25 passage;

(**C**) Group of N-AFS17 of 5 and 25 passages(p);

**(D)** Group of N-KYOU of 5 and 25 passages(p);

**(E)** Group of neural cultures (N-KYOU, N-AFS17, N-DP, N-DYP0730) of 5 passage.

**C**

**D**

**E**

The ordinate axis is the fluorescence level of the antibody measured as the ratio of the antibody staining index to the DAPI nuclear staining index (see methods). The length of the whiskers is limited to a maximum of 1.5 times the interquartile range. Cases marked "p < 0.05" showed statistically significant differences by the Mann-Whitney test.

**
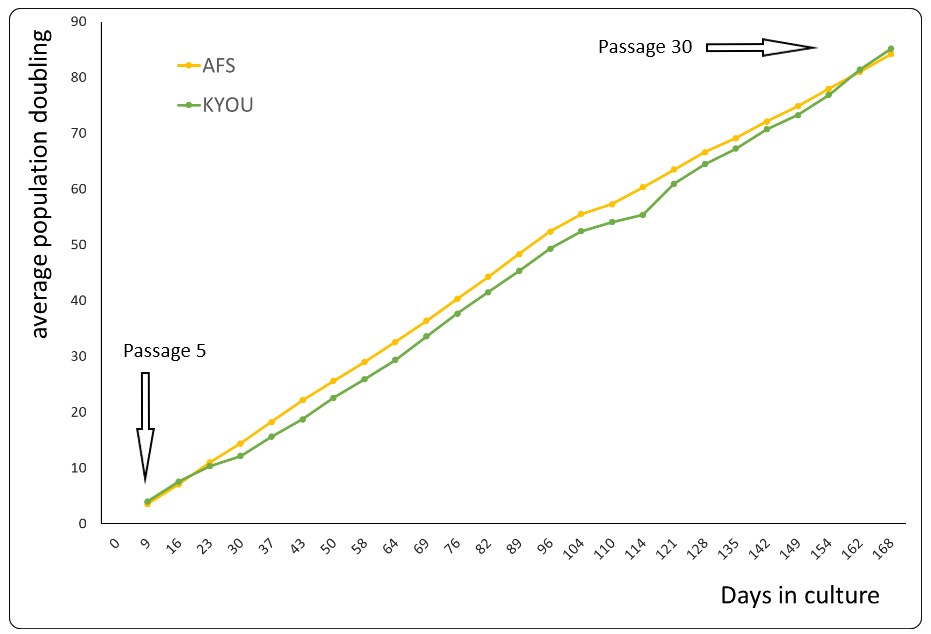
**

**Suppl. Fig. 6.** Proliferation rates of neural stem cell cultures (derived from IPSC-KYOU and IPSC-AFS17) during 5-30 passages.


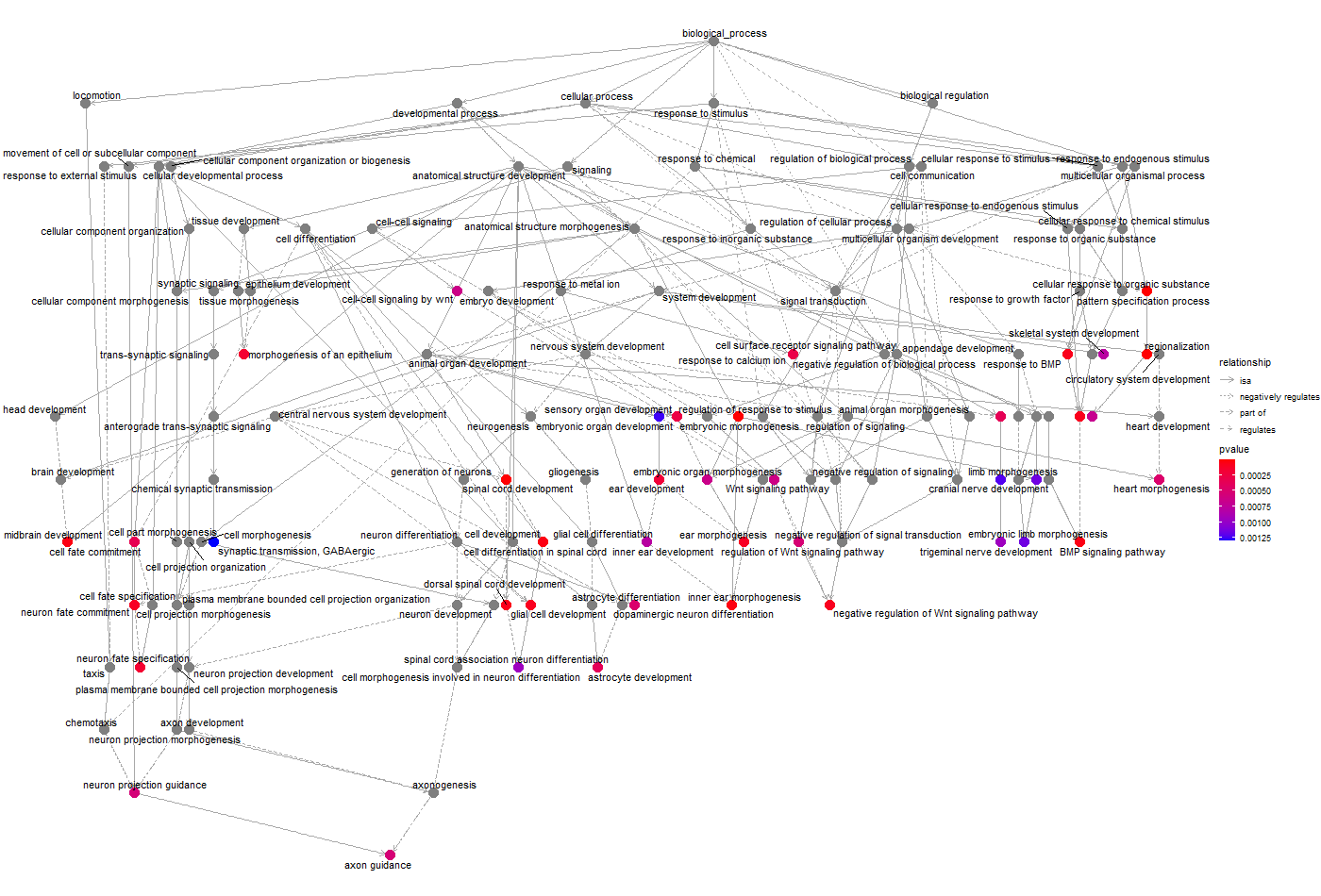


**Suppl. Fig.7** Graph of enriched ontologies from bulk transcriptome analysis of KYOU and AFS17 neural cultures


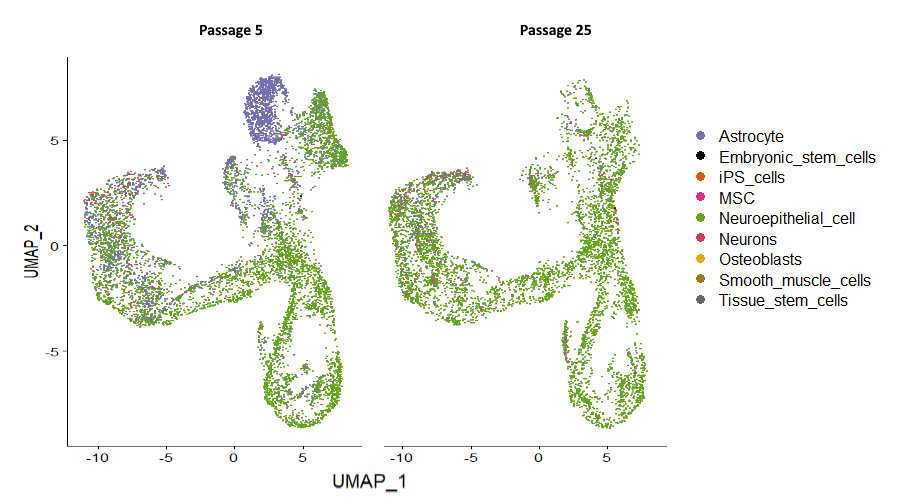


**Suppl. Fig.8.** Automated SingleR annotation of cells from different passages.


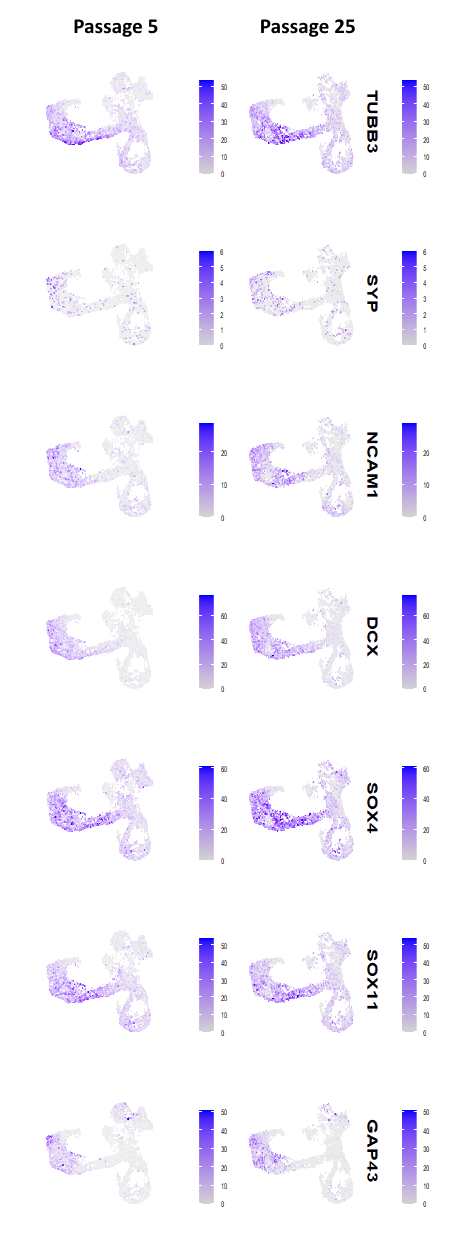


**Suppl. Fig.9.** Expression of neuron-specific genes on UMAP plots, at different passages.


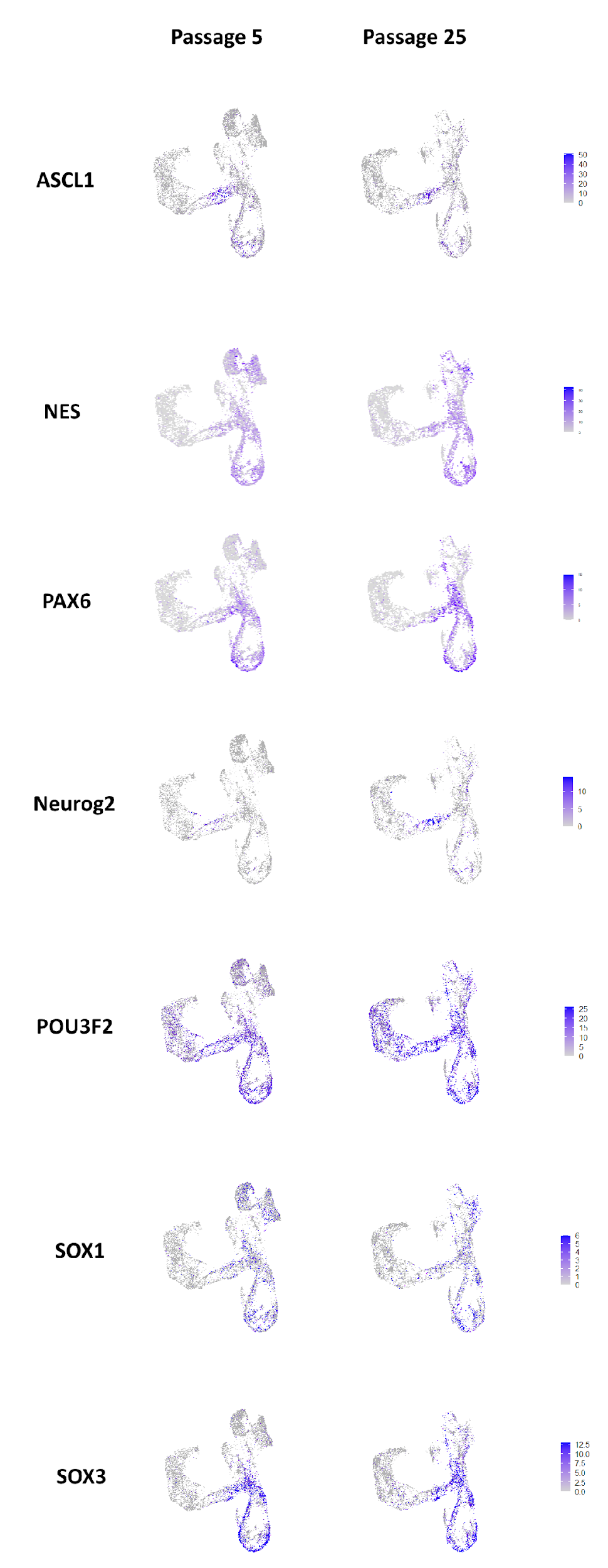


**Suppl. Fig.10.** Expression of neuron progenitor specific genes on UMAP plots, at different passages.


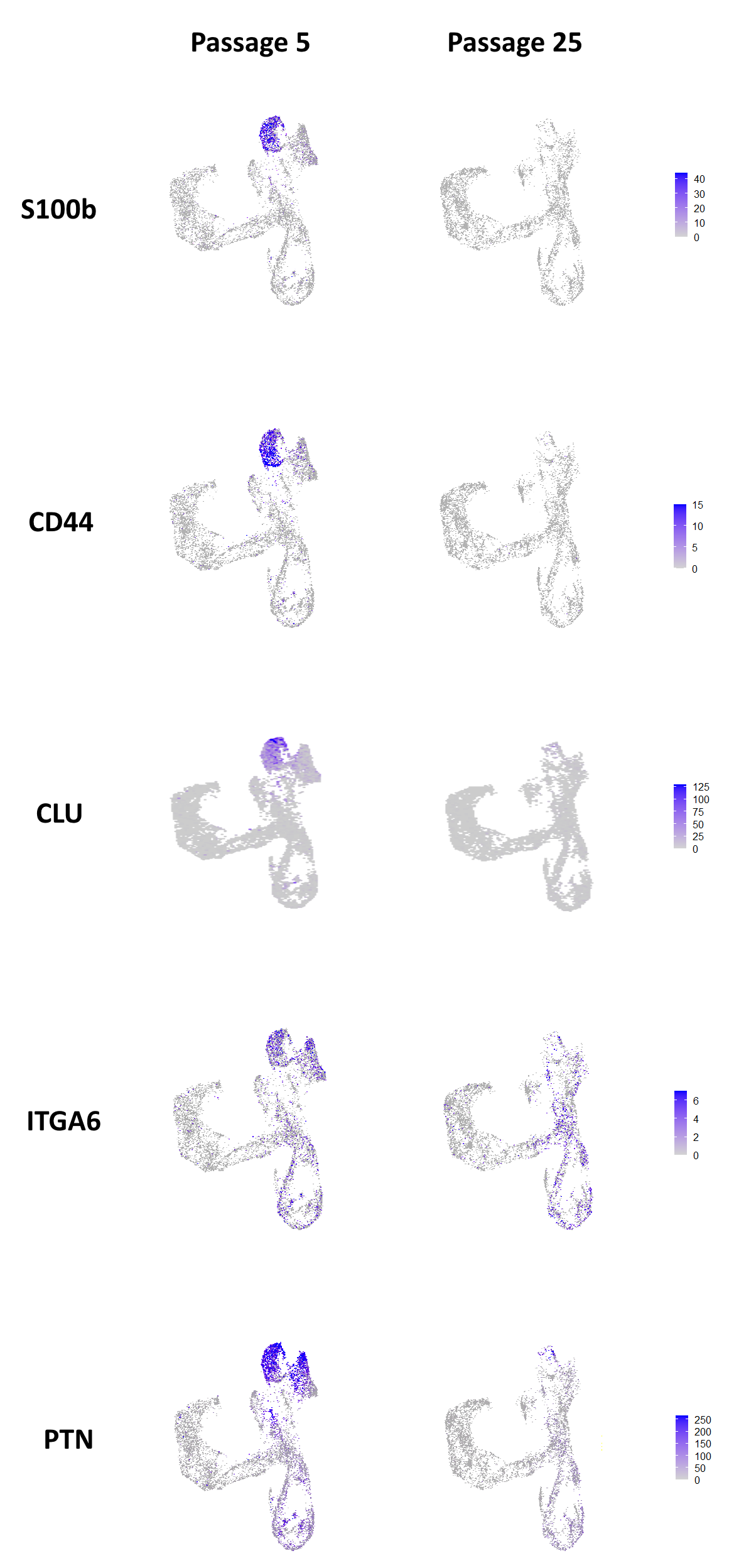


**Suppl. Fig.11** Expression of glial-specific genes on UMAP plots, at different passages.


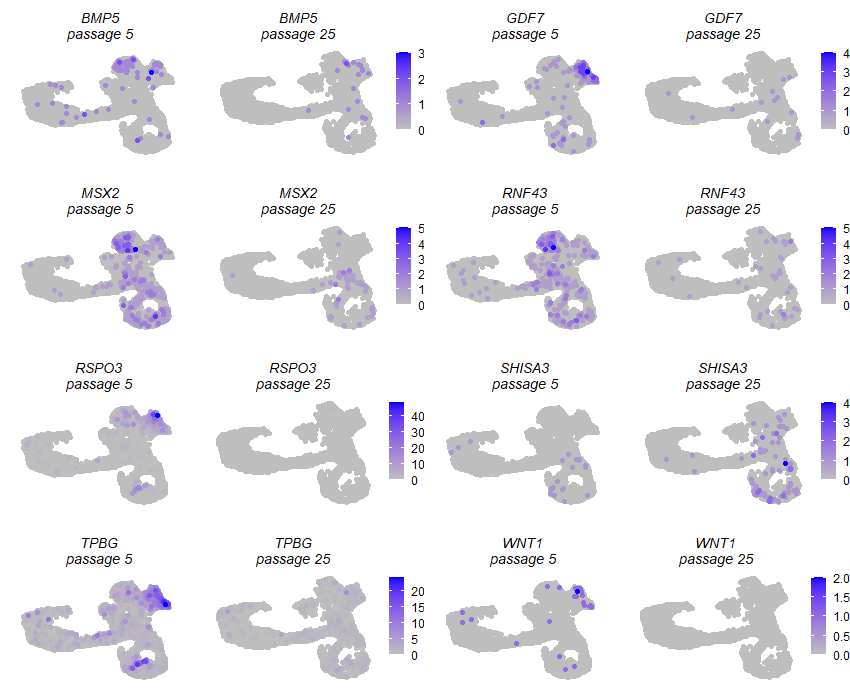


**Suppl. Fig.12.** Cell-type specific expression of common DEGs involved in WNT/BMP pathways in the KYOU scRNAseq experiment.


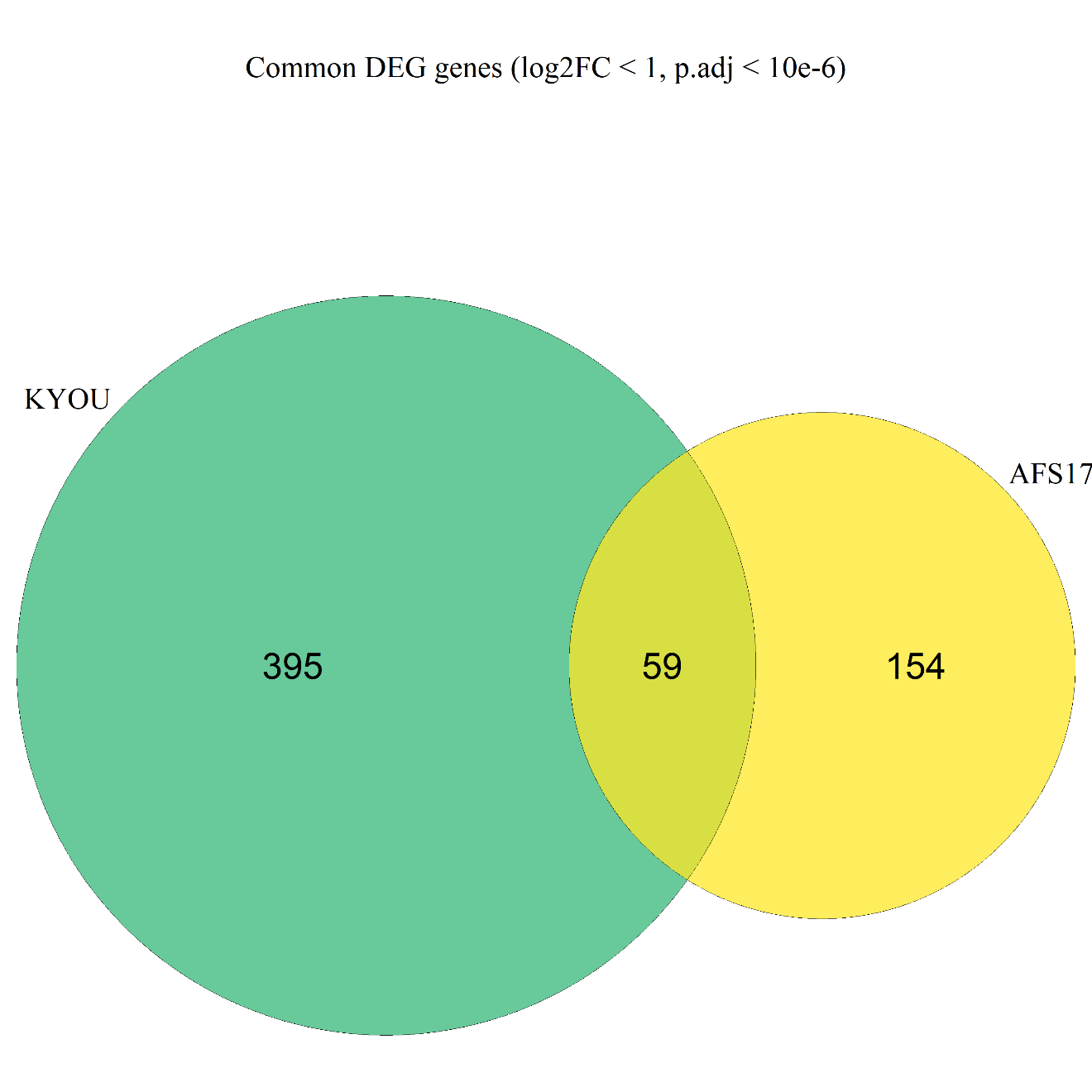


**Suppl. Fig.13.** Vienne daigram showing the overlap of common differentially expressed genes (DEGs), in an experiment with long-term culture of NS-KYOU and NS-AFS17.
